# Extricating human tumour immune alterations from tissue inflammation

**DOI:** 10.1038/s41586-022-04718-w

**Published:** 2022-05-11

**Authors:** Florian Mair, Jami R. Erickson, Marie Frutoso, Andrew J. Konecny, Evan Greene, Valentin Voillet, Nicholas J. Maurice, Anthony Rongvaux, Douglas Dixon, Brittany Barber, Raphael Gottardo, Martin Prlic

**Affiliations:** 1 Fred Hutchinson Cancer Research Center, Vaccine and Infectious Disease Division, Seattle, WA USA; 2 Department of Immunology, University of Washington, Seattle, WA USA; 3 Cape Town HVTN Immunology Laboratory, Hutchinson Centre Research Institute of South Africa, NPC (HCRISA), Cape Town, South Africa; 4 Molecular and Cellular Biology Graduate Program, University of Washington, Seattle, WA USA; 5 Fred Hutchinson Cancer Research Center, Clinical Research Division, Seattle, WA USA; 6 Department of Periodontics, School of Dentistry, University of Washington, Seattle, WA USA; 7 Department of Otolaryngology-Head and Neck Surgery, University of Washington, Seattle, WA USA; 8 Department of Statistics, University of Washington, Seattle, WA USA; 9 Department of Global Health, University of Washington, Seattle, WA USA; 10 Present Address: Department of Periodontics, University of Tennessee Health Science Center, College of Dentistry, Memphis, TN USA; 11 Present Address: University of Lausanne and Lausanne University Hospital, Switzerland, Lausanne, Switzerland

**Keywords:** Tumour immunology, Inflammation, Adaptive immunity, Immunotherapy

## Abstract

Immunotherapies have achieved remarkable successes in the treatment of cancer, but major challenges remain^1,2^. An inherent weakness of current treatment approaches is that therapeutically targeted pathways are not restricted to tumours, but are also found in other tissue microenvironments, complicating treatment^3,4^. Despite great efforts to define inflammatory processes in the tumour microenvironment, the understanding of tumour-unique immune alterations is limited by a knowledge gap regarding the immune cell populations in inflamed human tissues. Here, in an effort to identify such tumour-enriched immune alterations, we used complementary single-cell analysis approaches to interrogate the immune infiltrate in human head and neck squamous cell carcinomas and site-matched non-malignant, inflamed tissues. Our analysis revealed a large overlap in the composition and phenotype of immune cells in tumour and inflamed tissues. Computational analysis identified tumour-enriched immune cell interactions, one of which yields a large population of regulatory T (T_reg_) cells that is highly enriched in the tumour and uniquely identified among all haematopoietically-derived cells in blood and tissue by co-expression of ICOS and IL-1 receptor type 1 (IL1R1). We provide evidence that these intratumoural IL1R1^+^ T_reg_ cells had responded to antigen recently and demonstrate that they are clonally expanded with superior suppressive function compared with IL1R1^−^ T_reg_ cells. In addition to identifying extensive immunological congruence between inflamed tissues and tumours as well as tumour-specific changes with direct disease relevance, our work also provides a blueprint for extricating disease-specific changes from general inflammation-associated patterns.

## Abstract (web summary)

Complementary single-cell approaches show that a population of regulatory T cells co-expressing ICOS and IL-1 receptor type 1 is highly enriched in tumours but not in site-matched inflamed non-malignant tissue.

## Main

Antigen-presenting cells (APCs) and T cells residing in non-lymphoid tissues adapt distinct phenotypic and functional properties relative to their circulating counterparts in the peripheral blood^5–7^. These immune cells are also present in many solid tumour types, where they are thought to be critical determinants of tumour development and disease outcome^1,8^. One hallmark of immune-infiltrated human tumour tissues is the presence of an inflammatory microenvironment—this has been extensively scrutinized during the past decade^3,9^. However, since there is a paucity of studies on human non-malignant, inflamed tissues, it remains unclear which immune cell subsets and signalling pathways in the human tumour microenvironment are distinct from general inflammatory processes.

One of the best studied immune populations in tumour tissues is functionally exhausted (dysfunctional) T cells and T_reg_ cells, both of which are considered pivotal for inefficient anti-tumour immune responses^10,11^. These T cell subsets express immuno-inhibitory molecules such as programmed death 1 (PD-1) or cytotoxic T-lymphocyte-associated antigen 4 (CTLA4), which are the targets of various immunotherapeutic approaches^12^. However, expression of PD-1 and CTLA4 is not limited to tumour-infiltrating T cells and is also found on T cells in non-malignant tissues during homeostasis and inflammation^6,13^.

Notably, the effector program of T cells and their expression of immuno-regulatory molecules is closely linked to the function of (APCs), including dendritic cells, macrophages and other monocyte-derived cells^14^. APCs integrate tissue-specific and inflammation-dependent cues from the tissue environment, and can enhance or suppress local T cell responses^15^. Thus, functional alteration of APCs in the human tumour microenvironment has been suggested as an additional promising therapeutic target^16,17^.

We hypothesized that comparing the human tumour microenvironment with non-malignant, inflamed tissues could identify tumour-unique immune alterations that are distinct from general inflammatory responses. We thus combined several single-cell analysis pipelines to generate a comprehensive immune landscape of human head and neck squamous cell carcinoma (HNSCC) with site-matched non-malignant inflamed tissues from the oral cavity. Our data revealed substantial congruence of the immune phenotypes between these tissue groups. Computational analysis approaches identified tumour-specific changes in subsets of activated APCs and T_reg_ cells, including predicted major histocompatibility complex (MHC)–T cell receptor (TCR) and IL-1–IL1R signalling. Follow-up experiments confirmed these computational predictions: IL1R1^+^ T_reg_ cells in the tumour showed substantial clonal expansion, superior immunosuppressive function and hallmarks of recent TCR stimulation. Finally, this T_reg_ population could be identified among all haematopoietic cells by the combined expression of IL1R1 and ICOS, thus providing a unique opportunity to specifically target a large population of intratumoural T_reg_ cells.

## Phenotypic congruence of OM and HNSCC

Surgery is typically the first line of treatment for HNSCC, of which oral and oropharyngeal squamous cell carcinoma are subsites^18^. Non-malignant inflamed oral mucosal (OM) tissues (typically without prior anti-inflammatory treatment) from oral surgeries served as our reference. Together, this enabled us to compare the immune infiltrate of human inflamed with that of tumour tissues without therapeutic interventions as a confounding variable (sample list in Supplementary Table 1).

**Fig. 1:**
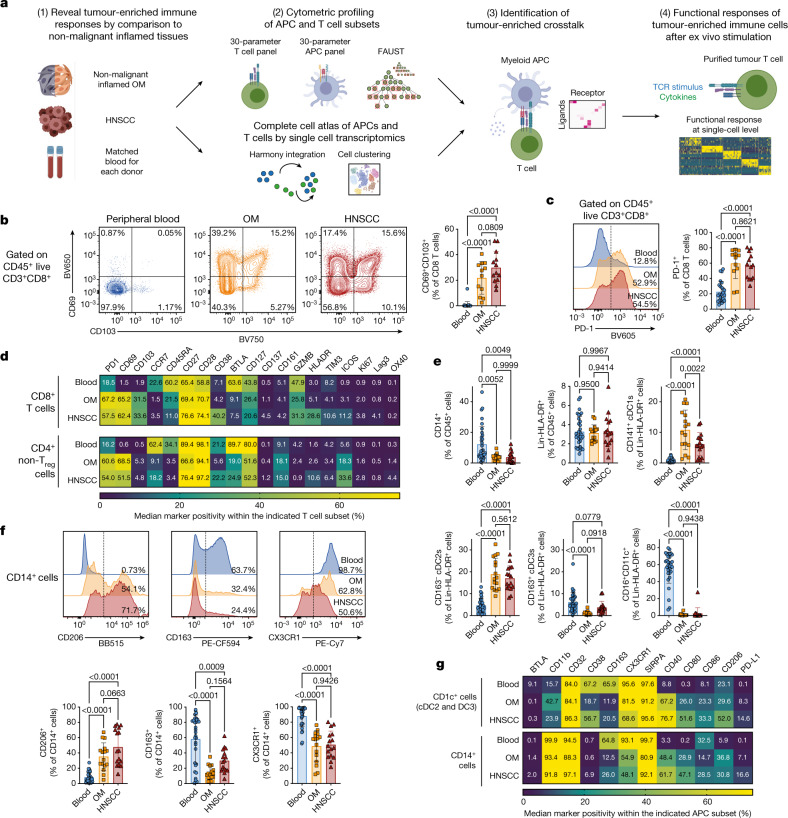
Similar immune phenotypes in inflamed non-malignant OM tissues and HNSCC. **a**, Overview of experimental strategy. **b**, Representative plots and quantification for CD69 and CD103 on CD8^+^ T cells from peripheral blood (blue), OM (orange) and HNSCC (red). **c**, Representative plots and quantification for PD-1 expression on CD8^+^ T cells. **d**, Heat map showing the expression pattern for all the indicated molecules within CD8^+^ T cells (top) and CD4^+^ helper T cells (without CD25^+^CD127^−^ T_reg_ cells, bottom) across peripheral blood, OM and HNSCC. **e**, Quantification of the indicated antigen-presenting cell (APC) populations. **f**, Representative histograms and quantification for CD206, CD163 and CX3CR1 on CD14^+^ cells. **g**, Heat map representing the expression pattern for all the indicated molecules within CD1c^+^ cDC2s and cDC3s (top) and CD14^+^ cells (bottom). All summary graphs are represented as mean ± s.d. (*n* = 12 for OM and *n* = 13 for HNSCC samples for T cell data, *n* = 16 for OM and HNSCC for APC data). Statistical analyses were performed using one-way ANOVA with Tukey’s multiple comparisons test.

**Extended Data Fig. 1:**
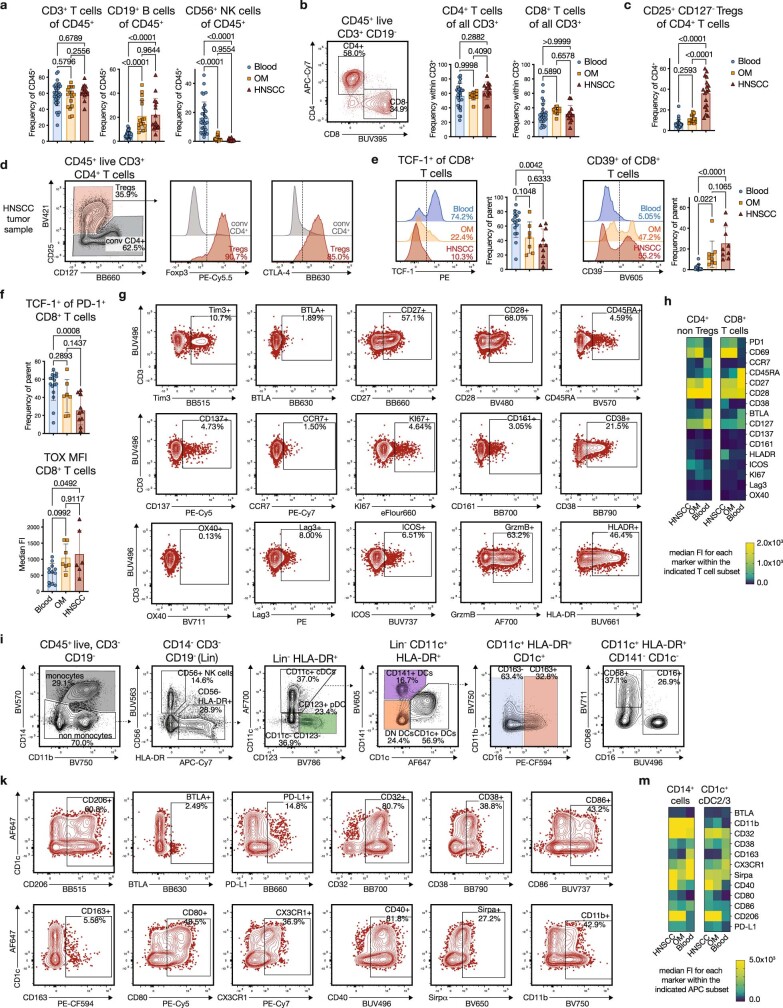
Additional immune subset quantifications and representative flow cytometry data (related to main Figure 1). (a) Quantification of CD3+ T cells, CD19+ B cells and CD56+ NK cells across the different tissue sources (blue: peripheral blood, orange: OM, red: HNSCC). (b) Frequency of CD4+ and CD8+ T cells within the CD3+ T cell compartment. (c) Quantification of CD4+ CD25+ CD127- regulatory T cells (Tregs) in the different tissue samples. (d) Intranuclear staining for Foxp3 and CTLA-4 on a representative HNSCC sample to confirm that CD25+ CD127- cells are bonafide Foxp3+ Tregs (red histograms: CD4+ CD25+ CD127- cells, grey histograms: CD4+ CD25- CD127+/- cells). (e) Representative histograms and quantification for the expression for TCF-1 (left, n = 7 for OM and n = 10 for HNSCC) and CD39 (right, n = 9) on CD8+ T cells. (f) Quantification for TCF-1 specifically on PD-1+ CD8+ T cells (left) and for the MFI of the transcription factor TOX on CD8+ T cells (right). (n = 7 for OM and n = 6 for HNSCC) (g) Staining patterns for phenotyping markers in the high-dimensional T cell panel, pregated on live CD3+ CD8+ T cells (HNSCC). Positivity cut-offs were the same for all samples, except where shifts in staining patterns based on density distributions indicated the need for adjustments. GrzmB staining showed donor-specific shifts, and Tim3 was impacted by autofluorescence in some donors. (h) Heatmap showing the median fluorescence intensities (MFI) for all the indicated molecules within CD8+ cytotoxic T cells (right) and CD4+ conv T cells (without CD25+ CD127- Tregs, left) across peripheral blood, OM, and HNSCC. This heatmap matches main Figure 1d, but shows MFIs instead of percentages. (i) Representative gating strategy for the identification of canonical antigen-presenting cell (APC) subsets in HNSCC. Plots are concatenated from three individual donors. (k) Staining patterns for all phenotyping markers in the high-dimensional APC panel shown on a representative HNSCC sample, pregated on live CD11c+ HLA-DR+ conventional DCs. Positivity cut-offs were left the same for all samples, except where shifts in staining patterns based on density distributions indicated adjustments. PD-L2 (on BV421) and CD85k (BV480) were excluded from all analyses because of significant variability due to autofluorescence between donors/experimental runs (data not shown). (m) Heatmap showing the median fluorescence intensities (MFI) for all the listed molecules within CD1c+ cDC2s/DC3s (right) and CD14+ cells (left). This heatmap matches main Figure 1g, but shows MFIs instead of percentages. Statistical analyses were performed using one-way ANOVA with Tukey’s multiple comparisons.

First, we extensively catalogued the immune landscape in OM and HNSCC tissues and matched blood by using 2 flow cytometry panels comprising 30 parameters (Fig. 1a, Supplementary Tables 2, 3) (adapted from ref. ^19^). The frequency of CD3^+^ T cells, CD19^+^ B cells and CD56^+^ natural killer (NK) cells among total CD45^+^ live cells as well as the CD4/CD8 ratio was essentially equivalent between OM and HNSCC tissues (Extended Data Fig. 1a, b). However, we observed a significant increase of CD4^+^CD25^+^CD127^−^Foxp3^+^ T_reg_ cells in HNSCC compared with OM tissues^20^ (Extended Data Fig. 1c, d).

Recent findings suggest that cytotoxic CD8^+^ T cells with a tissue-resident memory phenotype can be a principal predictor of tumour progression^8,21,22^. The expression patterns of the tissue residency markers CD69 and CD103 were very similar between OM and HNSCC tissues (Fig. 1b). PD-1, a biomarker of exhausted T cells^23^ was expressed by approximately 50% of total CD8^+^ T cells in both OM and HNSCC tissue samples (Fig. 1c). The transcription factors TCF1 and TOX, as well as CD39 showed similar expression patterns in OM- and HNSCC-infiltrating CD8^+^ T cells (Extended Data Fig. 1e, f). The same was true for the majority of markers for CD4^+^ and CD8^+^ T cells across both tissues (Fig. 1d, Extended Data Fig. 1g, h).

Next, we quantified subsets of APCs in the tumour microenvironment: CD14^+^ monocyte/macrophage-like cells, CD11c^+^CD141^+^ cross-presenting type 1 classical dendritic cells (cDC1s), CD11c^+^CD1c^+^CD163^−^ cDC2s, CD11c^+^CD1c^+^CD163^+^ DC3s (previously referred to as inflammatory dendritic cells^24–26^) and CD16^+^ non-conventional monocytes (Fig. 1e, Extended Data Fig. 1i). Whereas the relative abundance of CD14^+^ cells and total CD14^−^CD3^−^CD19^−^ (hereafter referred to as Lin^−^) HLADR^+^ cells was indistinguishable between OM and HNSCC tissues, we noted a slight decrease in the frequency of CD141^+^ cDC1s in HNSCC^27^ (Fig. 1e). cDC2s, DC3s and CD16^+^ cells were present in OM and HNSCC tissues with similar frequencies (Fig. 1e). Of note, we observed similar expression patterns for the mannose receptor CD206 (commonly used as a marker for alternatively activated, M2 macrophages) on CD14^+^ cells across the different tissues (Fig. 1f), indicating that M2-like phenotypes are not a specific hallmark of the tumour microenvironment^28^. A comparison of all biomarkers showed that tissue-infiltrating APC subsets were phenotypically relatively similar between OM and HNSCC tissues (Fig. 1f, g, Extended Data Fig. 1k–m). Together, these data indicate that the immune infiltrates in inflamed OM and HNSCC show substantial congruence in composition and phenotype.

**Extended Data Fig. 2:**
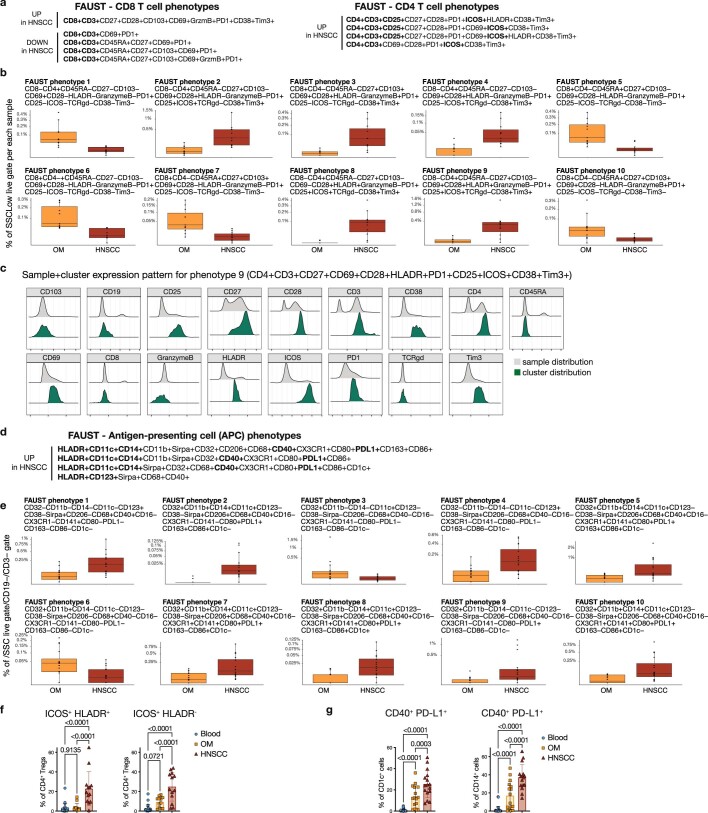
Computational analysis using FAUST for the T cell and APC panels (related to main Figure 1). (a) and (b) The top 10 T cell phenotypes showing differential abundance between OM (orange) and HNSCC (red) samples as identified by FAUST (FDR-adjusted level below 0.05). Population frequency is relative to the CD45+ live gate. Negative markers are not listed, all phenotypes are CD3+. (c) Example ridge plots of the marker distribution for T cell phenotype #9 (CD4+ CD3+ CD27+ CD69+ CD28+ HLADR+ PD1+ CD25+ ICOS+ CD38+ Tim3+) from a representative HNSCC sample. Grey histogram denotes marker distribution across all cells, green on the selected FAUST cluster. (d) and (e) A selection from the top 20 myeloid APC phenotypes showing differential abundance between OM (orange) and HNSCC (red) samples as identified by FAUST (FDR-adjusted level below 0.05). Population frequency is relative to the CD45+ live CD3- CD19- gate. Negative markers are not listed. (f) Simplifying the FAUST discovered Treg phenotype to just two markers, HLA-DR and ICOS. Summary plots show the relative frequency of ICOS+ HLADR+ cells (left) and ICOS+HLADR- cells (right) in the CD4+ CD25+ CD127- Treg compartment across blood, OM and HNSCC (n = 13). (g) Simplifying the FAUST discovered APC phenotype to just two markers, CD40 and PD-L1. Summary plots show the relative frequency of CD40+ PD-L1+ cells on CD1c+ DCs (left) and CD14+ cells (right) in blood, OM and HNSCC (n = 16). For the FAUST box plots, the lower bound of the box is the 25^th^ percentile (q25), center is the median, upper bound is the 75^th^ percentile (q75). The lower whisker is 1.5*interquartile below the q25, and the upper whisker is 1.5*interquartile above q75. Y-axis is shown on a square-root scale. Statistical analyses in (f) and (g) were performed using one-way ANOVA with Tukey’s multiple comparisons, and summary graphs are represented as mean ± SD.

To identify specific immune subset differences, we performed computational analysis using full annotation using shape-constrained trees (FAUST)^29^, a machine learning algorithm that discovers and annotates statistically relevant cellular phenotypes in an unsupervised manner. For the T cell panel, FAUST identified a single subcluster of CD8^+^ T cells, and four CD4^+^ T_reg_ (CD25^+^ CD127^−^) phenotypes marked by expression of ICOS with combinations of PD-1, TIM3 and HLA-DR as being enriched in HNSCC (Extended Data Fig. 2a–c). For the APC panel, FAUST identified several tumour-enriched phenotypes of CD14^+^ cells, cDC2s and cDC3s marked by co-expression of CD40 and PD-L1 (Extended Data Fig. 2d, e, g), resembling an activated APC phenotype. To further interrogate congruencies and differences in these immune phenotypes, we next used a single-cell RNA-sequencing (scRNA-seq) approach.

## Tumour-enriched cytokine modules in APCs

**Fig. 2:**
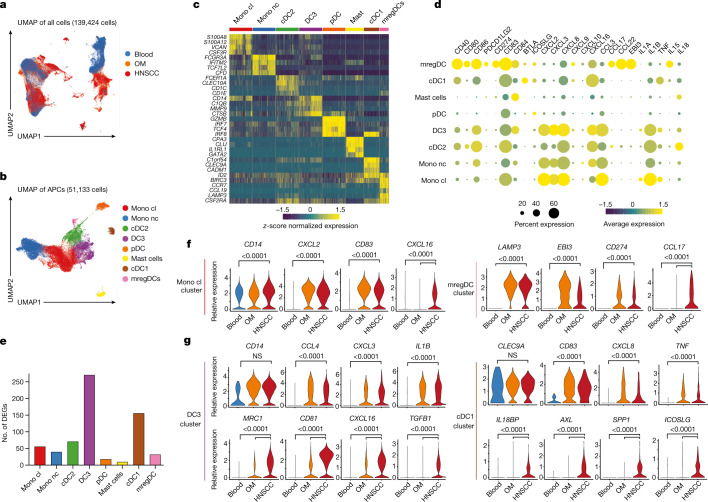
Comprehensive scRNA-seq analysis of OM and HNSCC immune infiltrates. **a**, UMAP of the combined scRNA-seq data after quality filtering and Harmony integration, coloured by tissue origin (more details in Extended Data Fig. 3). **b**, UMAP plot of the APC populations after subsetting and reclustering, coloured by cluster. Mono cl, classical monocyte; Mono nc, non-classical monocyte. **c**, Key DEGs in each APC cluster. **d**, Scaled dot plot showing the transcript expression across APC clusters from combined OM and HNSCC data (excluding blood). **e**, Number of DEGs between HNSCC and OM-derived cells per APC cluster as determined by MAST. **f**, Violin plots showing the expression of selected transcripts for the monocyte cluster (left) and the mregDC cluster (right). **g**, Violin plots showing the expression of selected transcripts for the DC3 cluster (left) and the cDC1 cluster (right). All graphs are showing combined data for *n* = 4 for OM samples and *n* = 4 for HNSCC samples, with a total of 139,424 cells after filtering for quality control criteria. Violin plots show adjusted *P*-values (Bonferroni correction) as calculated by the Seurat implementation of MAST.

**Extended Data Fig. 3:**
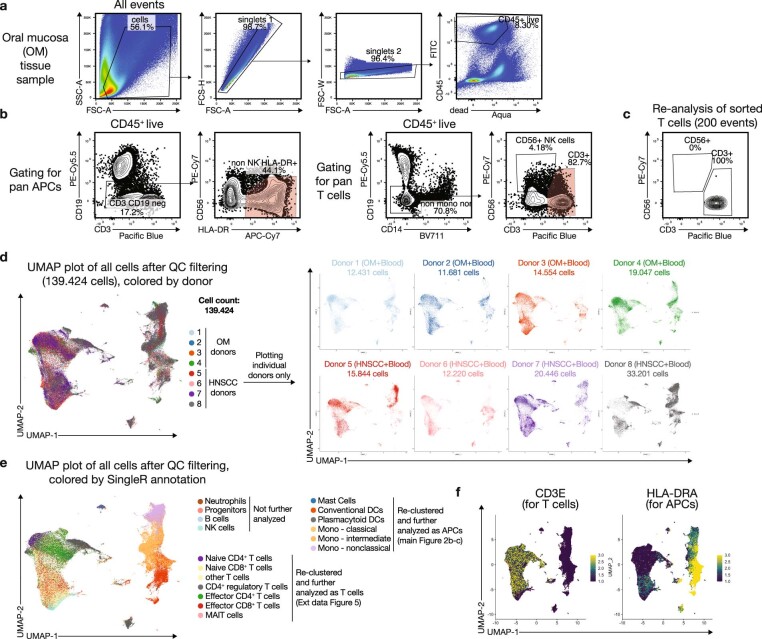
Gating strategy for the sorts and additional plots for the scRNA-seq data (related to main Figure 2). (a) General gating of a representative OM tissue samples for CD45+ live singlets for the WTA 10x scRNA-seq experiments. (b) Gating strategy used for sorting the pan APC population and for pan T cells for WTA 10x scRNA-seq experiments. Red shaded gates were sorted. For some experiments (data not shown) MR1-Tetramer+ MAIT cells and CD56+ NK cells were sorted separately. (c) Representative re-analysis of a fraction of sorted pan T cells before loading onto the 10x Chromium controller. (d) UMAP plots of the combined scRNA-seq data after QC filtering (see Github script) and Harmony integration, colored by donor. A total of 139.424 cells is shown. Right plots depict individual donors separately, showing that cells from each donor distribute across the entire plot. Of note, for some donors only the T cell or the APC population could be sorted. (e) UMAP plots of the combined scRNA-seq data after QC filtering and Harmony integration, colored by simplified cell type calling derived from SingleR. The populations indicated in the legend were used for re-clustering and more in-depth analysis of the APC population (main Figure 2b) and T cells (Extended Data Fig. 5), respectively. (f) UMAP plots show heatmap overlays for CD3E and HLA-DRA transcripts to highlight the main lineages.

To ensure analysis of rare T cell and APC subsets, we sorted pan CD3^+^ T cells and Lin^−^HLA-DR^+^ cells from fresh OM and HNSCC tissues with matched blood (Extended Data Fig. 3a–c). After filtering for quality control and data integration using Harmony^30^, we obtained approximately 140,000 cells from 8 donors (Extended Data Fig. 3d). After dimensionality reduction using uniform manifold approximation and projection^31^ (UMAP) and cellular annotation using SingleR^32^, canonical T cell and APC populations separated clearly on the UMAP plot (Extended Data Fig. 3e). Cells derived from OM and HNSCC mostly grouped together, but separate from peripheral blood (Fig. 2a), in line with the phenotypic overlap found in our flow cytometry data.

**Extended Data Fig. 4:**
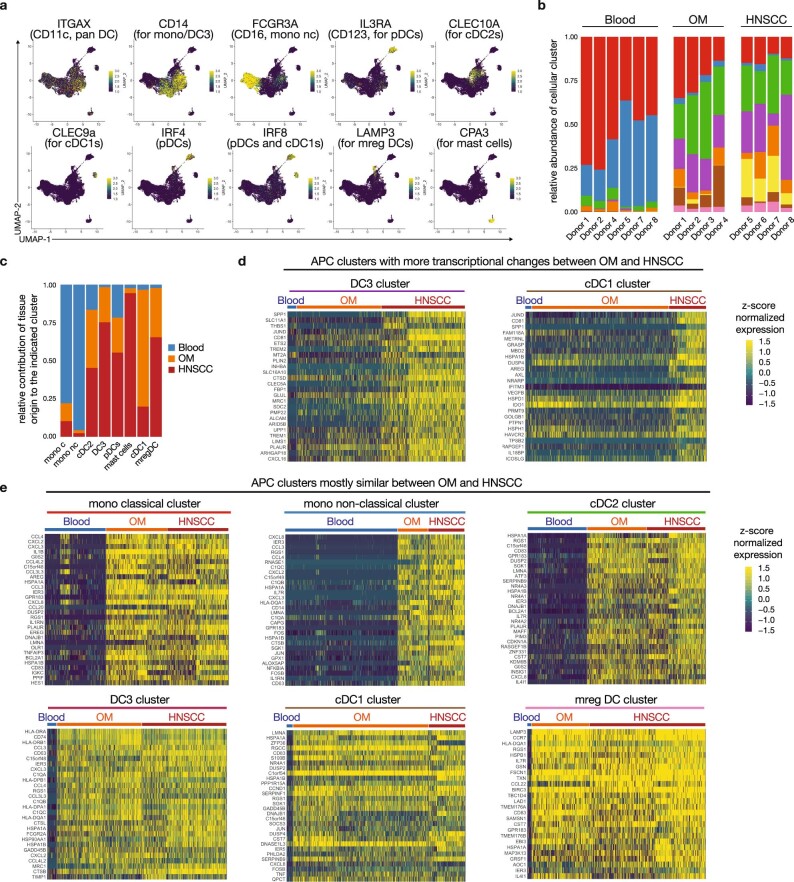
Additional analyses of the scRNA-seq data for the APC populations (related to main Figure 2). (a) Heatmap overlays for the expression of key lineage transcripts on the UMAP plot for the APC population (see main Figure 2b). (b) Relative abundance of the APC clusters across donors and tissue origin. (c) Relative contribution of each tissue source to the indicated APC cluster (colors match the cluster description in main Fig. 2b). (d) Heatmaps showing the top 30 transcripts that were differentially expressed in HNSCC for the DC3 cluster (left) and cDC1 cluster (right), which are the two clusters showing the largest number of DE genes between OM and HNSCC (see main Figure 2e). (e) Heatmaps showing the top 30 transcripts that were shared between OM and HNSCC, but differentially expressed from matched peripheral blood, for each of the indicated APC clusters (mono classical, mono non-classical, cDC2s, DC3s, cDC1s and mreg DCs). For (d) and (e), the APC clusters identified in the scRNA-seq data (main Figure 2b) were subsetted, and either the genes shared between OM and HNSCC cells, or genes differentially expressed between HNSCC and OM cells were identified by the Seurat implementation of MAST (see material and methods and Github script for additional details).

Next, we re-clustered APCs and mapped these populations to established lineages (Fig. 2b, Extended Data Fig. 4a). We also found a population of HLA-DR-expressing mast cells mainly in HNSCC tissues, marked by the signature genes *CLU* (encoding mast cell carboxypeptidase A) and *GATA2* (Fig. 2c, Extended Data Fig. 4a). In line with our flow cytometry data, cDC1s were reduced in HNSCC tissues, while the other APC subsets appeared similarly distributed in both tissues across all donors (Extended Data Fig. 4b, c), including a population expressing high levels of *CCR7*, *CCL19* and CSF2RA (encoding the GM-CSF receptor) (Fig. 2c), resembling mature dendritic cells enriched in immunoregulatory molecules^33^ (mregDCs). When we analysed the abundance of transcripts of key co-regulatory genes, chemokines and cytokines across all APC subsets from OM and HNSCC (Fig. 2d), we noted that shared modules of chemokine transcripts were detected in the monocyte, cDC2 and DC3 clusters, whereas mregDCs were the dominant CCL17- and CCL22-expressing population. CXCL8 and CXCL16 expression was detected in all subsets, albeit to varying degrees.

To determine whether the transcriptional activity of these APC clusters changed in HNSCC relative to the inflamed OM, we identified differentially expressed genes (DEGs) in each cluster using model-based analysis of single-cell transcriptomics^34^ (MAST). MAST revealed a pronounced alteration of DC3 and cDC1 transcriptomes in HNSCC compared with OM (that is, 150–250 genes), whereas the remaining APC clusters showed more congruent profiles (Fig. 2e). Manually selected genes that were either shared between tissues or enriched in HNSCC are shown in Fig. 2f, g, and heat maps are shown in Extended Data Fig. 4d, e. Overall, this comparison highlights that altered transcriptional signatures in HNSCC tissues are detected mainly in cDC1 and DC3 subsets and include differential expression of gene encoding cytokines (*TGFB1* and *IL18BP*) and co-receptors (*ICOSLG*).

**Extended Data Fig. 5:**
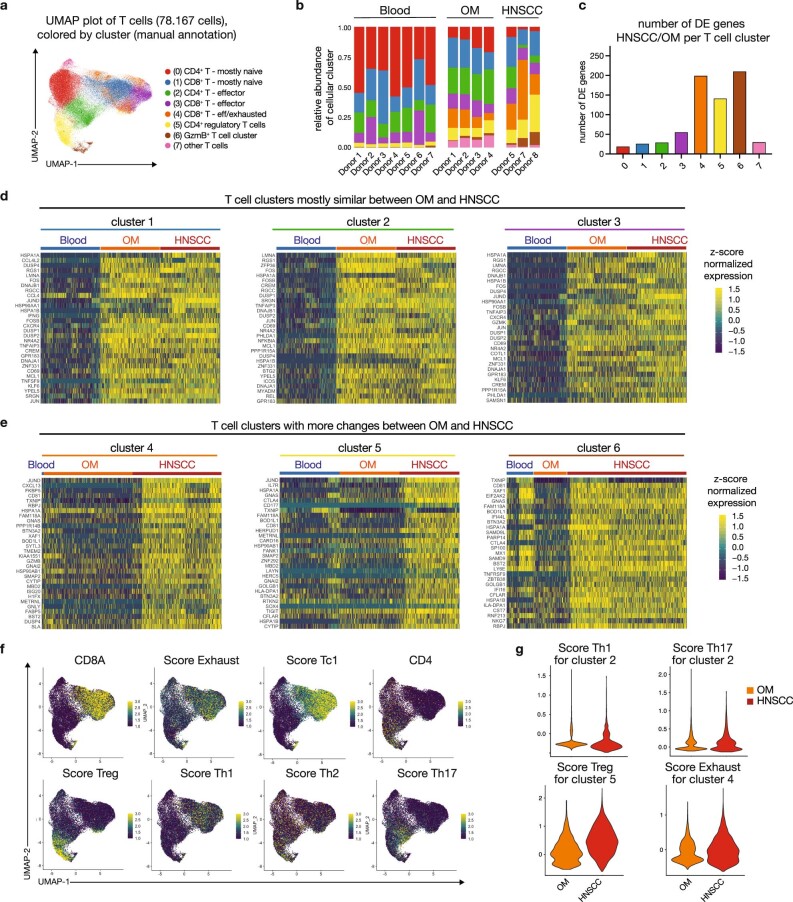
Additional analyses of the scRNA-seq data for T cell populations (related to main Figure 2). (a) UMAP plot of the T cell populations after subsetting and reclustering, colored by cluster. (b) Relative cluster abundance across donors and tissue origin (color code is the same as in panel a) (c) Number of DE genes between HNSCC and OM-derived cells per T cell cluster as determined by MAST. (d) Heatmaps showing the top 30 transcripts that were shared between OM and HNSCC, but differentially expressed from matched peripheral blood, for each of the indicated T cell clusters. (e) Heatmaps showing the top 30 transcripts that were differentially expressed in HNSCC for the T cell clusters 4–6, which were the two clusters showing the largest number of DE genes between OM and HNSCC. (f) Heatmap overlays showing the indicated lineage transcripts and T cell scores (see material and methods) on the UMAP plot. (g) Violin plots depicting the relative T cell score in the indicated HNSCC (red) vs OM-derived (orange) T cell clusters.

Finally, re-clustering of the T cells suggested that there were eight transcriptionally separate populations, which we annotated manually (Extended Data Fig. 5a). Again, cluster distribution across donors and tissues was remarkably constant, with the HNSCC samples showing an expansion of the CD4^+^ T_reg_ cluster (Extended Data Fig. 5b). We noted that the number of DEGs between HNSCC and OM-infiltrating cells showed the largest change in two CD8^+^ T cell clusters and in the T_reg_ cluster (Extended Data Fig. 5c), with the other clusters being rather similar (Extended Data Fig. 5d). Of note, the DEGs in these two CD8 T cell clusters were associated with cytotoxic properties (Extended Data Fig. 5e). Calculating T cell lineage scores for the T cell clusters further highlighted the increase in the T_reg_ signature (Extended Data Fig. 5f, g), in line with our flow cytometry data.

## Tumour-enriched APC-T cell crosstalk

**Fig. 3:**
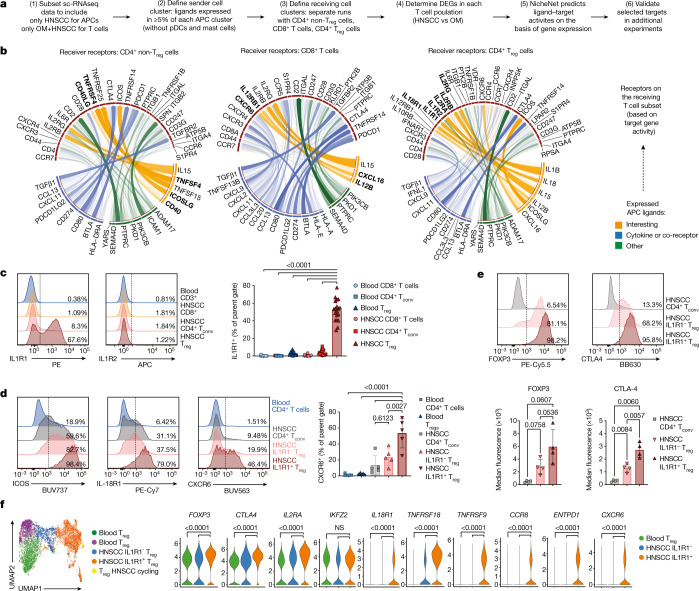
NicheNet analysis predicts tumour-enriched APC-T cell crosstalk. **a**, The NicheNet workflow was applied to the scRNA-seq data shown in Fig. 2. **b**, Circos plots showing the top ligand–receptor pairs identified by NicheNet. Transparency of the connection represents the interaction strength. APC ligands are on the bottom, TCRs are on top. **c**, Representative plots and quantification for the surface protein expression of IL1R1 (*n* = 19). **d**, Representative plots showing the expression of ICOS, IL-18R1 and the chemokine receptor CXCR6 on the indicated T cell subsets. Right, quantification for CXCR6 (*n* = 5). **e**, Representative plots (top) and mean fluorescence intensity (bottom) for FOXP3 and CTLA4 on T cell subsets from HNSCC (*n* = 4). **f**, UMAP plot of T_reg_ cells sorted from blood and tumour of *n* = 3 donors with HNSCC after targeted transcriptomics, coloured by cluster. Violin plots show the expression of selected transcripts across T_reg_ clusters. All summary graphs are represented as mean ± s.d. Statistical analyses of cytometry data was performed using one-way ANOVA with Tukey’s multiple comparisons test, analysis of targeted transcriptomics used the Seurat implementation of MAST (adjusted *P*-values after Bonferroni correction).

**Extended Data Fig. 6:**
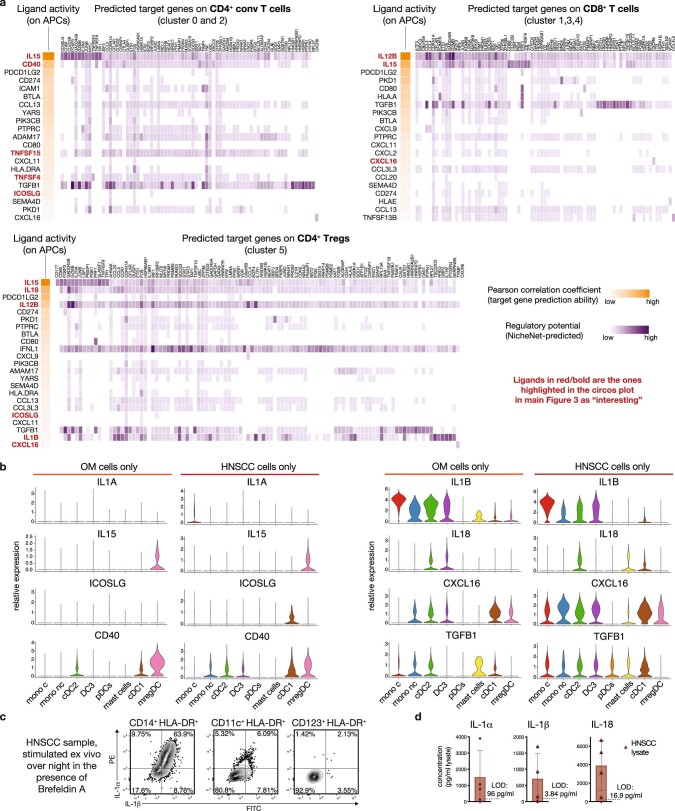
Additional plots related to the NicheNet predictions (related to main Figure 3). (a) Plots derived from the NicheNet analysis showing the predicted ligand activity (orange) for the top ligands (as ranked by Pearson correlation coefficient), and the predicted target genes (purple) for all three separate NicheNet runs: sender APCs + receiver cells CD4+ conv T cell clusters (upper left plots), sender APCs + receiver CD8+ T cell clusters (upper right plots) and sender APCs + receiver CD4+ Treg cluster (lower plots). The full script utilized for the NicheNet analysis is available on Github (see material and methods). The ligands that are highlighted in main Figure 3b as “interesting” are highlighted in red/bold in this panel here. (b) The APC clusters from main Figure 2b were subsetted to contain only OM or HNSCC-derived cells, and Violin plots depict relative expression of the indicated ligand transcripts across all APC clusters either in OM-infiltrating cells (left columns) or HNSCC infiltrating cells (right columns). The ligands here match the ones highlighted as “interesting” in main Figure 3b. (c) Representative plots showing the protein expression for the cytokines IL-1α and IL-1β after ex vivo culture of bulk HNSCC-derived APCs in the presence of Brefeldin A, followed by intracellular cytokine staining. (d) Concentration of IL-1α and IL-1β and IL-18 as measured by Luminex analysis in flash-frozen HNSCC samples (n = 4). LOD: limit of detection.

To better understand the relevance of the immune alterations that we had observed, we investigated potential tumour-specific cross-talk between T cells and APCs using NicheNet^35^ (workflow in Fig. 3a). We set the APC clusters derived from scRNA-seq (excluding plasmacytoid dendritic cells (pDCs) and mast cells) as the sender population, and the CD4^+^ conventional T cell, CD8^+^ T cell and CD4^+^ T_reg_ clusters as separate receiver populations. For each T cell subset, we focused our analysis on the top 20 ligand–receptor pairs (Fig. 3b, Extended Data Fig. 6a). Several signalling axes were shared by T cell subsets in the tumour microenvironment (for example, the ligands CD80, CD274 and PDCD1LG2, and the cytokines TGFβ1 and IL-15). NicheNet predicted several enriched ligand–receptor interactions between APCs and T_reg_ cells in HNSCC: ICOS ligand (ICOSLG) via ICOS, IL-18 via the IL18-R1 and IL-1B via the IL-1 receptors type 1 and type 2 (Fig. 3b, right). Of note, when we assessed which APC population expressed transcripts for these ligands, some were found in multiple subsets in both OM and HNSCC (for example, *TGFΒ1*, *IL1B* and *CXCL16*), whereas *ICOSLG* was found only on cDC1s from the HNSCC (Fig. 2g, Extended Data Fig. 6b).

Since our flow cytometry and scRNA-seq approaches both indicated T_reg_ changes in the HNSCC, we further explored the predicted T_reg_–APC interactions. We first tested whether the receptors predicted by NicheNet were present as proteins, and found that IL1R1 was expressed specifically by tumour-infiltrating T_reg_ cells, but not by tumour-infiltrating CD4^+^ T cells or CD8^+^ T cells, nor by T cells in the peripheral blood (Fig. 3c). Notably, up to 70% of the T_reg_ cells expressed IL1R1, whereas IL1R2, a decoy receptor for IL-1 signalling, was detectable on less than 2% of cells. Nearly all IL1R1^+^ T_reg_ cells co-expressed ICOS and IL-18R1 in conjunction with higher levels of the chemokine receptor CXCR6 (Fig. 3d), matching the NicheNet predictions. Finally, both IL1R1^+^ and IL1R1^−^ intratumoural T_reg_ cells expressed FOXP3, but CTLA4 expression was significantly increased only in the IL1R1^+^ fraction (Fig. 3e).

We next addressed whether intratumoural APCs had the capacity to express IL-1. Following ex vivo culture, a majority of HNSCC-derived CD14^+^ cells and some pan cDCs expressed IL-1β and IL-1α protein (Extended Data Fig. 6c). Analysis of flash-frozen whole-tumour lysate revealed substantial levels of IL-1α, IL-1β and IL-18 (Extended Data Fig. 6d). Together, these data indicate that intratumoural IL-1 is probably available for IL1R1^+^ T_reg_ cells, but also raise the question of the biological and clinical relevance of the IL1R1^+^ T_reg_ population.

To determine how IL1R1^+^ T_reg_ cells differ from their IL1R1^−^ counterparts, we used a targeted transcriptomics approach^36^ to measure the expression of genes with known relevance for immune function (Supplementary Table 4). Intratumoural IL1R1^+^ and IL1R1^−^ T_reg_ cells (from *n* = 3 tumours) formed separate clusters (Fig. 3f, orange and blue, respectively), distinct from blood T_reg_ cells. More than 50 transcripts were selectively enriched in the IL1R1^+^ cluster, including *TNFRSF18* (which encodes GITR) and TNFRSF9 (which encodes 4-1BB), which has been suggested as a pan-cancer T_reg_ target^37^ (Fig. 3f). Overall, these data suggest that IL1R1^+^ T_reg_ cells represent a transcriptionally distinct population of intratumoural T_reg_ cells.

## IL1R1^+^ T_reg_ cells are highly suppressive

**Fig. 4:**
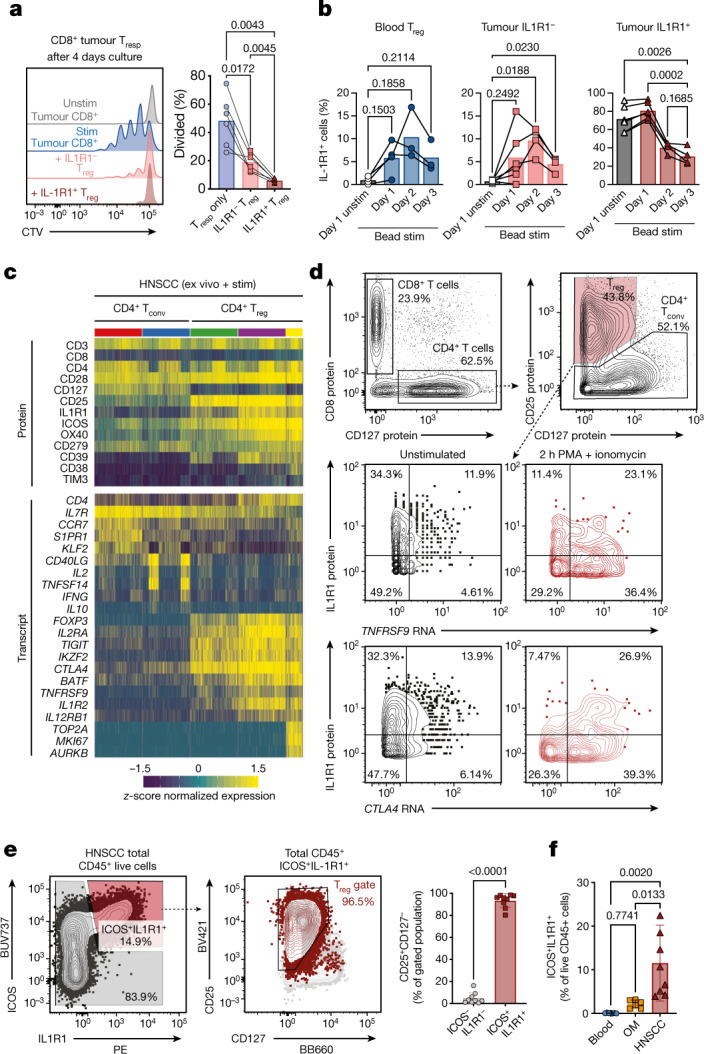
IL1R1-expressing T_reg_ cells represent a functionally distinct population. **a**, Proliferation of HNSCC-derived CD8^+^ T responder (T_resp_) cells (*n* = 6) in an in vitro suppression assay with IL1R1^−^ T_reg_ cells (light red) and IL1R1^+^ T_reg_ cells (dark red). Representative histograms show dilution of Cell Trace Violet. Stim, stimulated; unstim, unstimulated. **b**, Expression kinetics of IL1R1 after in vitro culture in the presence of anti-CD3/CD28/CD2 beads for T_reg_ cells sorted from peripheral blood (left, *n* = 3), IL1R1^−^ T_reg_ cells (middle, *n* = 4) and IL1R1^+^ T_reg_ cells (right, *n* = 5) from HNSCC. **c**, Tumour-infiltrating T cells from two donors with HNSCC after performing short-term stimulation and targeted transcriptomics with AbSeq (Extended Data Fig. 8). Heat maps show top differentially expressed proteins (top) and transcripts (bottom) across the selected clusters. **d**, TNFRSF9 and CTLA4 transcript expression by T_reg_ cells left unstimulated (left) and after short-term stimulation with PMA and ionomcyin (right). The *y*-axis shows IL1R1 protein expression. **e**, Representative plots and quantification (*n* = 9) showing that within total CD45^+^ cells in HNSCC nearly all ICOS^+^ IL1R1^+^ cells are T_reg_ cells. **f**, Quantification of total ICOS^+^ IL1R1^+^ cells in peripheral blood (*n* = 7), OM (*n* = 6) and HNSCC samples (*n* = 8). All summary graphs are represented as mean ± s.d. Statistical analyses were performed using one-way ANOVA with Tukey’s multiple comparisons test or using a two-tailed paired *t*-test (**e**).

**Extended Data Fig. 7:**
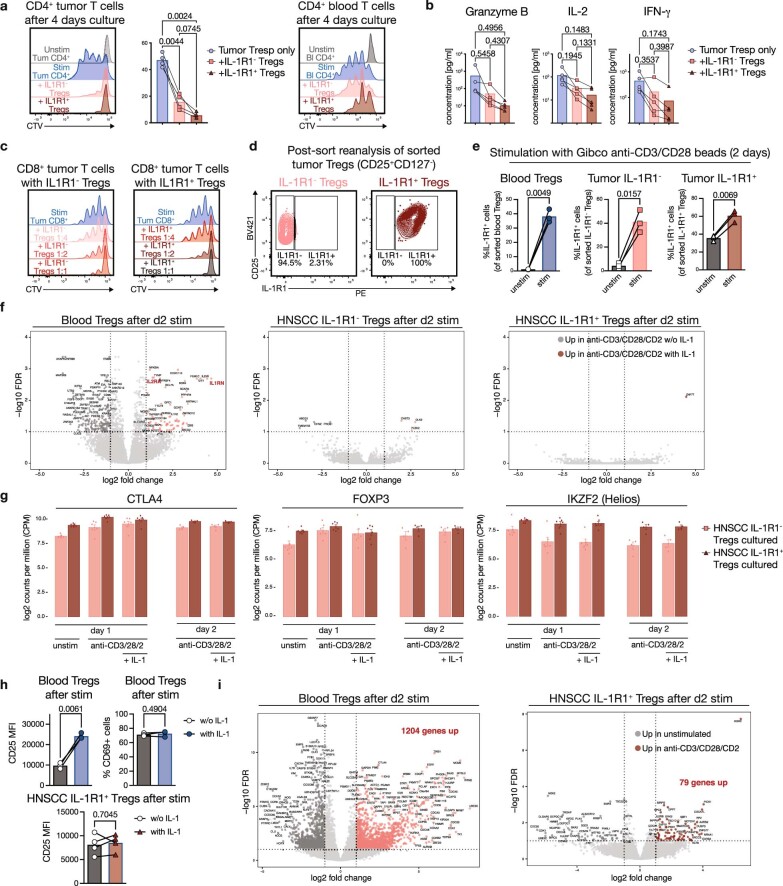
Additional data from Treg suppression and stimulation assays (related to main Figure 4). (a) Proliferation of HNSCC-derived CD4+ T responder cells from tumor (left) or from blood (right) in an in vitro suppression assay with IL-1R1- Tregs (light red) and IL-1R1+ Tregs (dark red) from tumor. Representative histograms show dilution of Cell Trace Violet (CTV) after 4 days. n = 4. (b) Concentration of Granzyme B, IL-2 and IFN-g in the culture supernatants of the 4-day suppression assays shown in main Figure 4a. (c) Proliferation of HNSCC-derived CD8+ T responder cells in an in vitro suppression assay with titrated amounts of IL-1R1- Tregs (left plot) and IL-1R1+ Tregs (right plot). Histograms show CTV dilution after 4 days of culture with Treg-to-Teff ratios of 1:4, 1:2 and 1:1 (top to bottom). (d) Plots depict representative post-sort purity of HNSCC-Treg populations used for the stimulation experiments in main Figure 4b. (e) Expression of IL-1R1 after two days of in vitro culture either unstimulated or in the presence of Gibco anti-CD3/CD28 beads for Tregs sorted from peripheral blood (blue), IL-1R1- (light red) and IL-1R1+ Tregs (dark red) from HNSCC. n = 3. (f) Volcano plots showing differential gene expression of SMART-Seq bulk RNAseq data (250 sorted cells) of blood Tregs, HNSCC IL-1R1- and IL-1R1+ after 2 days of culture with anti-CD3/CD28/CD2 beads with (light and dark red) or without IL-1 (grey). (g) Transcripts per million for FOXP3, Helios (IKZF2) and CTLA4 from the bulk RNAseq data for the indicated time points and culture conditions. n = 4 for the d2 time point, n = 6 for the d1 time point. (h) CD25 median fluorescence intensity (MFI) on the indicated Treg populations after 2 days of culture with anti-CD3/CD28/CD2 beads +/− IL-1. n = 3 for blood and n = 4 for the HNSCC samples. (i) Volcano plots showing differential gene expression of SMART-Seq bulk RNAseq data (250 sorted cells) of blood Tregs and HNSCC IL-1R1+ Tregs after 2 days either unstimulated or with anti-CD3/CD28/CD2 beads. Number of DE genes upregulated is highlighted in bold.

To directly assess the functional capacity of intratumoural IL1R1^+^ T_reg_ cells, we established classic suppression assays suitable for the low cell numbers from HNSCC tissues. We found that IL1R1^+^ T_reg_ cells were more effective than their IL1R1^−^ counterparts at suppressing proliferation of CD8^+^ (Fig. 4a) and CD4^+^ responder T (T_resp_) cells isolated from tumours as well as from peripheral blood (Extended Data Fig. 7a). Furthermore, we observed a decrease in the concentration of effector molecules in the culture supernatant in the presence of IL1R1^+^ T_reg_ cells (Extended Data Fig. 7b), and suppression was dependent on the ratio of T_reg_ cells to T_resp_ cells (Extended Data Fig. 7c).

Since NicheNet predicted that T_reg_ cells were receiving TCR signals (Fig. 3b), we tested whether a TCR signal is sufficient to induce IL1R1 expression. We sorted T_reg_ cells from peripheral blood, and IL1R1^−^ and IL1R1^+^ T_reg_ cells from HNSCC (Extended Data Fig. 7d). IL1R1 expression on blood T_reg_ cells and IL1R1^−^ tumour T_reg_ cells was induced after 24 h of stimulation with anti-CD3, anti-CD28 and anti-CD2 beads (anti-CD3/CD28/CD2 beads) (Fig. 4b). IL1R1 expression on IL1R1^+^ tumour T_reg_ cells was sustained on day 1, but declined by day 2 and day 3. Stimulation of T_reg_ cells with anti-CD3 and anti-CD28 coated beads optimized for T cell activation led to a more pronounced increase in IL1R1 expression (up to 50% of blood T_reg_ cells and IL1R1^-^ tumour T_reg_ cells, Extended Data Fig. 7e). These data indicate that a TCR combined with a costimulation signal is sufficient to induce IL1R1 expression.

To assess the functional impact of IL-1 signalling on T_reg_ cells, we performed bulk-RNA sequencing of blood T_reg_ cells and IL1R1^−^ and IL1R1^+^ HNSCC T_reg_ cells after culture with anti-CD3/CD28/CD2 beads with or without IL-1 for 2 days. The addition of IL-1 led to significant upregulation of more than 50 genes in T_reg_ cells isolated from blood, including *IL2RA*, but had minimal effects on the transcriptional profile of intratumoural T_reg_ cells (Extended Data Fig. 7f). Expression of *FOXP3*, *CTLA4* and IKZF2 (which encodes Helios) was unaffected by IL-1 signals across all T_reg_ populations, indicating stable maintenance of the T_reg_ phenotype (Extended Data Fig. 7g). The increase in *IL2RA* was mirrored by an increase of IL2RA (also known as CD25) protein expression (Extended Data Fig. 7h). A comparison of the transcriptional profiles of the T_reg_ populations after TCR stimulation revealed that both blood and HNSCC IL1R1^−^ T_reg_ cells showed high transcriptional activity (more than 900–1,200 transcripts were upregulated), whereas the changes in IL1R1^+^ T_reg_ cells were much less pronounced (only around 70 transcripts were upregulated; Extended Data Fig. 7i). Together, these data suggest that IL-1 may increase the suppressive function of T_reg_ cells that are not yet fully activated by increasing CD25 expression, whereas intratumoural IL1R1^+^ T_reg_ cells are already highly activated and less responsive to additional stimulatory signals.

**Extended Data Fig. 8:**
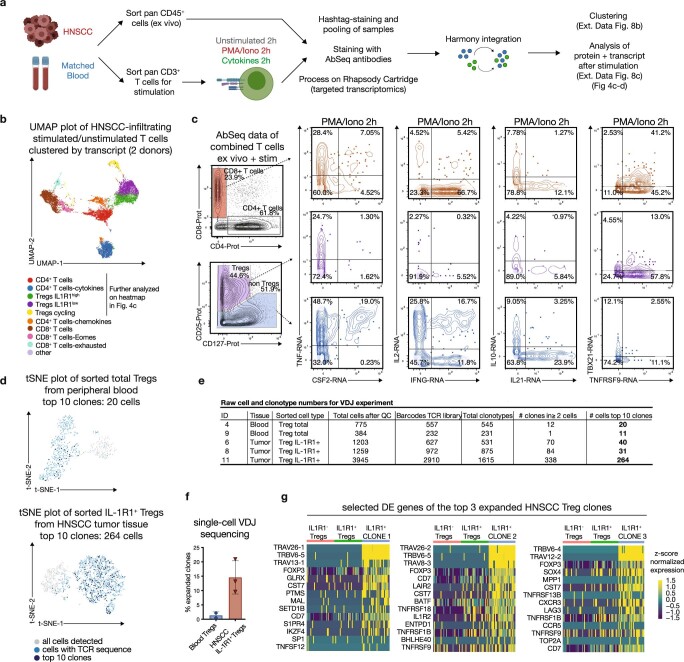
Additional analyses for the VDJ and targeted transcriptomics/Abseq data (related to main Figure 4). (a) Experimental outline for the targeted transcriptomics+AbSeq experiments (see also material and methods). Combined data set after Harmony integration consists of ex vivo CD45+ cells, unstimulated T cells, and PMA-Ionomycin stimulated T cells from two different donors. (b) UMAP plot of tumor-infiltrating T cells of two different HNSCC donors after targeted transcriptomics and AbSeq as described in (a), colored by clustering based on transcript. The highlighted clusters are shown in more detail in main Figure 4c. (c) Selected cytokine transcripts are shown on manually gated T cell subsets from HNSCC AbSeq data (populations as identified by surface protein). Red plots show CD8+ T cells, violet plots show CD25+ CD127- Tregs, and blue plots show CD25- CD4+ non Tregs (all after PMA/Iono for 2h). TBX21 encodes for T-bet, TNFRSF9 encodes for CD137 (4-1BB). (d) t-SNE plot of sorted Tregs from the peripheral blood (top) and tumor (bottom) from a single donor after VDJ sequencing (10x genomics 5’ v1 chemistry). All cells with a complete TCR sequence are marked in light blue, and the top 10 clonotypes are marked in dark blue (encompassing 20 cells in the blood, and 239 cells in the tumor). (e) Table showing the total cell counts, barcode counts and detected clonotypes after VDJ sequencing for the indicated samples (n = 3 for the HNSCC tumor samples, n = 2 for matched peripheral blood). Counts are derived from VDJ Loupe browser. (f) Expanded clones by single-cell VDJ sequencing within sorted IL-1R1+ Tregs from HNSCC tumors relative to total Tregs from matched peripheral blood. Every TCR sequence that was present in 2 cells or more was considered an expanded clone. (n = 2 for blood, n = 3 for HNSCC) (g) Differentially expressed (DE) genes for the top 3 expanded clones from one single donor. Heatmaps show a selection of the top DE genes as identified by MAST for each clone (right side of each heatmap) vs downsampled HNSCC IL1R1- Tregs and IL1R1+ Tregs.

To further assess the responsiveness of these T_reg_ cells to activating signals we used AbSeq for combined protein and transcript profiling of HNSCC-infiltrating T cells with and without short-term stimulation with phorbol myristate acetate (PMA) and ionomycin (Extended Data Fig. 8a). Focusing our analysis on CD4^+^ T cells and T_reg_ cells (Extended Data Fig. 8b, c), unsupervised clustering revealed three T_reg_ clusters: IL1R1^−^, IL1R1^+^ and an actively proliferating IL1R1^+^ T_reg_ cluster marked by *MKI67* and *TOP2A* (Fig 4c). T_reg_ cells stimulated with PMA and ionomcyin showed increased expression of *TNFRSF9* and *CTLA4* transcripts, and IL1R1^+^ T_reg_ cells showed the largest increase in CTLA4 protein expression (Fig. 4d). These data further underline the notion that IL1R1^+^ T_reg_ cells are activated and functional, while encompassing a subset that is actively proliferating. Thus, we next assessed TCR diversity of IL1R1^+^ T_reg_ cells using single-cell VDJ sequencing. We found that 10–20% of IL1R1^+^ T_reg_ cells in HNSCC tumours were clonally expanded, with the top 10 clones in the tumour making up more than 5% of the total number of cells recovered for some donors (Extended Data Fig. 8d–f). The top 3 expanded clones showed distinct gene expression profiles relative to the remaining cells—for example, enrichment for *TNFRSF9* (Extended Data Fig. 8g).

The data collected so far indicate that IL1R1 expression marks a highly suppressive and expanded subset of intratumoural T_reg_ cells. We therefore tested whether it was possible to uniquely identify these cells with a small set of biomarkers. We found that nearly all cells in the CD45^+^IL1R1^+^ICOS^+^ gate were T_reg_ cells (Fig. 4e). Thus, the combined expression of just two cell surface-expressed proteins—IL1R1 and ICOS—could uniquely identify these T_reg_ cells among all haematopoietic (CD45^+^) cells in HNSCC and blood. Finally, in a set of follow-up experiments we assessed whether IL1R1^+^ICOS^+^ T_reg_ cells were truly enriched in HNSCC over inflamed OM tissues and found that IL1R1^+^ICOS^+^ cells were significantly enriched in HNSCC compared with OM (Fig. 4f).

**Extended Data Fig. 9:**
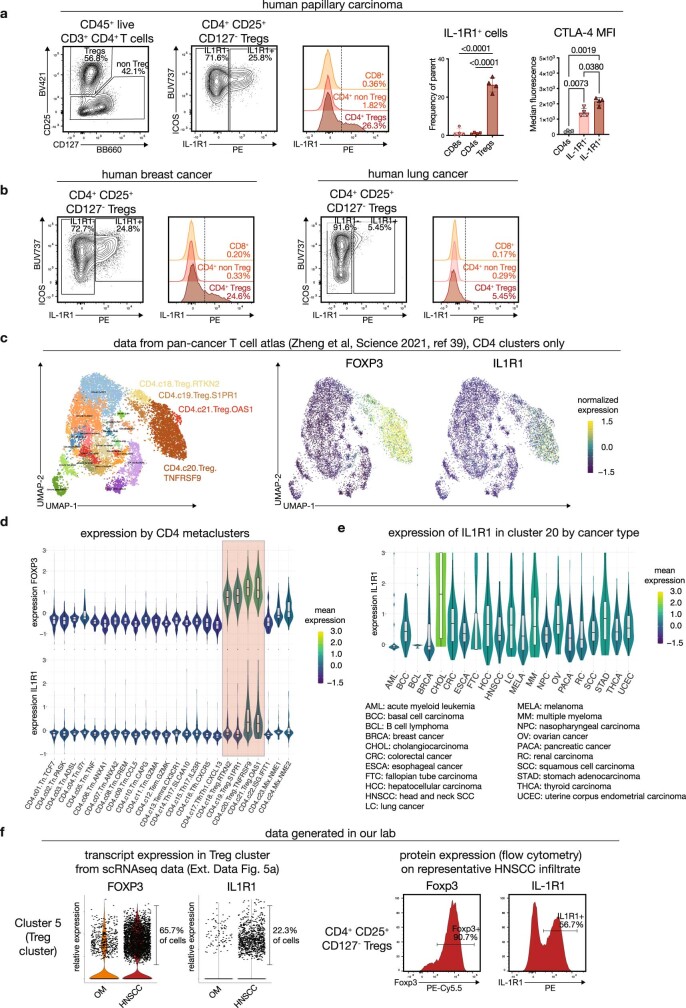
IL-1R1 expression in other cancer types (related to main Figure 4). (a) IL-1R1 expression is present on CD4+ CD25+ CD127- Tregs in tumor tissues from a cohort of human papillary carcinoma patients. Plot on the very right shows quantification of the MFI for CTLA-4 on the indicated populations (n = 4 donors, one-way ANOVA with Tukey’s multiple comparisons). (b) To assess IL-1R1 protein expression in additional tumor types, we were able to collect a single sample of human breast cancer (left) and human lung cancer (right). IL-1R1 expression is present on breast cancer Tregs, but only at very low level/absent on lung cancer Tregs. (c) UMAP plot of all CD4 T cell metaclusters. There are four Treg clusters, which are highlighted on the right side of the UMAP plot. Heatmap overlays on the right side show expression of the transcripts encoding FOXP3 and IL1R1. (d) Violin plots depicting expression of FOXP3 (top panel) and IL1R1 (lower panel) across all CD4 T cell clusters of the combined data set as annotated by Zheng et al. Shaded area highlights the Treg clusters. IL1R1 expression is primarily found in cluster 20 and 21. (e) Violin plots depicting expression of IL1R1 in cells from cluster 20 CD4.c20.Treg.TNFRSF9) across different tumor types. While expression appears to be absent in AML and BCL, varying levels of IL1R1 transcript are present across all other tumor types. (f) IL1R1 transcript expression in scRNA-seq data may underestimate protein expression. Left panel shows violin plots for expression of the indicated transcripts in the Treg cluster 5 of our combined scRNAseq data from OM and HNSCC (see Extended Data Fig. 5a), right histograms show protein expression for the same genes by flow cytometry on live CD4+ CD25+ CD127- Tregs on a representative HNSCC sample. Data in panels c-e are publicly available from the pan-cancer T cell atlas (containing 21 cancer types), Zheng et al, Science 2021 (ref. ^39^). Figures were generated using the Shiny app on http://cancer-pku.cn:3838/PanC_T. Per the authors’ description, box plot overlays show 25^th^ percentile (lower bound), median (center) and 75^th^ percentile (upper bound), whiskers extend +/− 1.5*interquartile range. Data in panel a-b and f are from our own tissue collections.

To determine whether IL1R1^+^ T_reg_ cells occur specifically in HNSCC, we analysed and found IL1R1^+^ T_reg_ cells in a small set of additional solid tumour types by flow cytometry (Extended Data Fig. 9a, b). Furthermore, we mined publicly available scRNA-seq datasets^38,39^, and found that among intratumoural T cells, expression of *IL1R1* transcript was largely restricted to T_reg_ cells (Extended Data Fig. 9c, d) and was detected across 19 different tumour types (Extended Data Fig. 9e, f). Together, these analyses indicate that IL1R1^+^ T_reg_ cells are not unique for HNSCC, but are also present to varying degrees in other solid tumours.

## Discussion

Overall, our data reveal that immune phenotypes typically associated with the human tumour microenvironment are also present in non-malignant, inflamed tissues. The expression pattern of PD-1 was essentially identical in T cells from both tumour tissues and non-malignant, inflamed tissues, which could offer an explanation for sometimes severe side-effects of systemic anti-PD-1 treatment^2,40^. Of note, PD-1 expression is typically considered to be driven by TCR signals, but is also upregulated by pro-inflammatory cytokines^41,42^, which may explain the high expression levels in inflamed tissues. Furthermore, our data indicate that mregDCs^33^ are also present in non-malignant, inflamed tissues with minimal transcriptional changes in tumour tissues.

**Extended Data Fig. 10:**
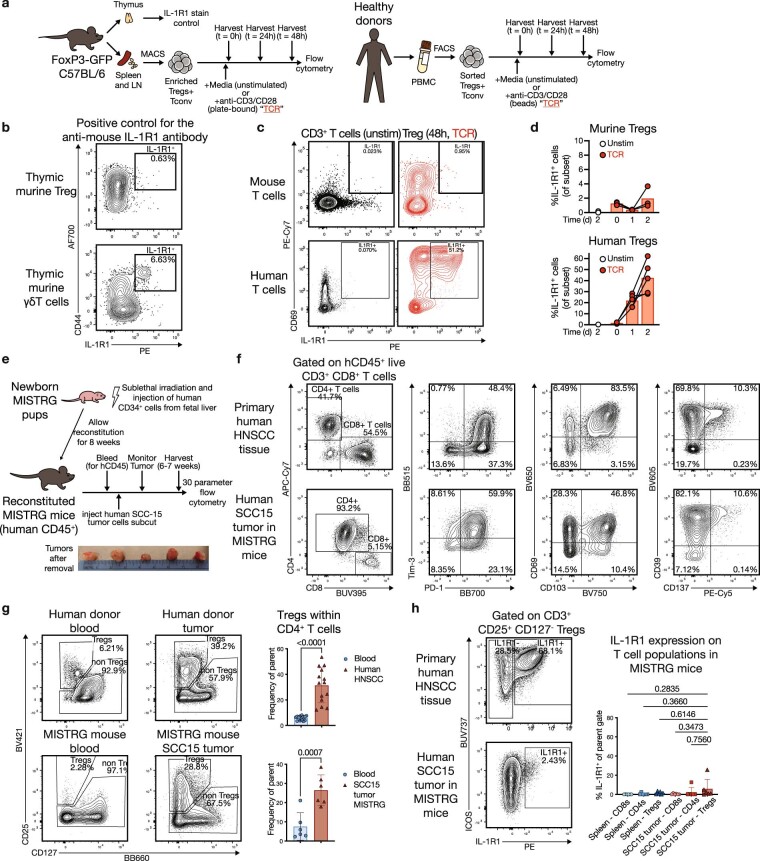
Differential expression kinetics of IL-1R1 in human cells, murine cells and a humanized mouse model. (a) Overview of stimulation experiments for murine (left) and human (right) purified Treg populations. (b) Murine thymic γδ T cells (lower plot) were used as a positive control to validate the anti-mouse IL-1R1-PE antibody signal. (c) Representative plots depicting IL-1R1 and CD69 expression on unstimulated (black) and TCR stimulated (red) Tregs after 48 h. (d) Quantification of IL-1R1 expression after stimulation for 1 or 2 days in murine (top panel, n = 3) and human Tregs (bottom panel, n = 5), highlighting the discrepancy in expression level and kinetics. (e) Overview and timeline of human SCC15-tumor experiment in humanized MISTRG mice. Bottom, photograph of 5 tumors after collection. (f) Representative flow cytometry plots showing similar expression patterns for a set of key T cell markers in primary human HNSCC biopsies (top) and SCC15 tumors in MISTRG mice reconstituted with human immune cells (bottom). (g) Representative plots and quantification showing a similar increase in Treg frequencies between human blood/HNSCC tumor tissue (top) and humanized mouse blood/SCC15 tumor tissue (bottom). n = 6 for the humanized mouse samples, and n = 14 for the human HNSCC samples. (h) Representative plots and quantification showing that IL-1R1 expression is detectable, but under-represented in the humanized mouse model (n = 5) compared to primary human HNSCC tissue (see main Figure 3c). Error bars represent mean +/- SD. Statistical analyses were performed using one-way ANOVA with Tukey’s multiple comparisons test for (h) or using a two-tailed paired t-test for (g).

NicheNet analysis of receptor–ligand interactions^35^ predicted that T_reg_ cells in the tumour actively received TCR signals and responded to IL-1 and IL-18. IL-18 has been implicated in inducing a tissue-repair program by secretion of amphiregulin from IL-18R^+^ T_reg_ cells^43^. Less is known about the effects of IL-1, traditionally considered a pro-inflammatory cytokine, on T_reg_ cells^44–46^. Whereas IL1R1 expression on T_reg_ cells has been reported in vitro^47^, our data revealed that a transcriptionally and functionally distinct subset of IL1R1^+^ T_reg_ cells is present in vivo in human HNSCC. Of note, we considered using the mouse model system for additional mechanistic studies, but mouse T_reg_ cells did not express IL1R1 in response to TCR-mediated stimulation (Extended Data Fig. 10a–d). We also tried to recapitulate the IL1R1^+^ T_reg_ phenotype in a humanized mouse model of squamous cell carcinoma (Extended Data Fig. 10e). The intratumoural immune infiltrate of the humanized mice was remarkably similar to that in primary human HNSCC tumours, including expression of PD-1, tissue-resident memory T (T_RM_) cell markers (Extended Data Fig. 10f) and increased T_reg_ infiltration (Extended Data Fig. 10g). However, we only observed low levels of IL1R1 expression by intratumoural T_reg_ cells in humanized mice (Extended Data Fig. 10h). One possible explanation for this difference could be that the tumour microenvironment in humanized mice is sterile, whereas HNSCC (and other human tumours) can contain a viable microbiome^48^. Overall, these data highlight how our human tissue comparison approach identified a T_reg_ population that is otherwise missed in various mouse models.

Of note, compared with IL1R1^−^ T_reg_ cells or CD4^+^ T cells, IL1R1^+^ T_reg_ cells expressed higher levels of CXCR6, which was recently reported to be critical for anti-tumour activity of cytotoxic T cells in a mouse model^49^. It is tempting to speculate that this chemokine–receptor pair could also regulate T_reg_ migration and co-localization with cytotoxic CD8^+^ T cells in the tumour, on the basis of the expression of the ligand CXCL16 in DC3s (Fig. 2g).

The depletion of tumour-infiltrating T_reg_ cells is considered a promising anti-tumour therapy^50–52^. However, therapeutic manipulation of T_reg_ cells in the tumour without affecting other T_reg_ populations has proved difficult. We show that the co-expression of IL1R1 and ICOS uniquely identifies an intratumoural T_reg_ population from all other haematopoietically-derived (CD45^+^) cells in the tumour or peripheral blood. This could provide a possible pathway for tumour-specific depletion of a large intratumoural T_reg_ population using bi-specific antibodies or logic-gated chimeric antigen receptor (CAR) T cells. Overall, our approach could serve as a blueprint to identify immunological congruencies and differences across other tissue types and disease states to improve our understanding of disease-specific processes.

## Methods

### Primary cells

The head and neck squamous cell carcinoma (HNSCC) tissue samples were obtained after informed consent from otherwise treatment-naïve patients undergoing surgical resection of their primary tumour, ensuring that the immune infiltrate was not influenced by prior therapeutic interventions such as radiotherapy. Inflamed OM tissue biopsies were obtained from individuals undergoing routine dental surgeries for a variety of inflammatory conditions such as periimplantitis, periodontitis or osseous surgery. Matched peripheral blood samples were collected from each tissue donor if possible. All study participants signed a written informed consent before inclusion in the study, and the protocols were approved by the institutional review board (IRB) at the Fred Hutchinson Cancer Research Center (IRB#6007-972 and IRB#8335). A detailed list of the samples and relevant procedure information, together with the panels and/or sequencing experiment performed is provided in Supplementary Table 1. Furthermore, cryopreserved peripheral blood mononuclear cells (PBMCs) from healthy controls (Seattle Area Control Cohort (SAC)) were obtained via the HIV Vaccine Trial network (HVTN) and used for titrations, panel development and as a longitudinal technical control for all flow cytometry acquisitions (data not shown). The human squamous cell carcinoma line SCC-15 was obtained and validated from ATCC (tested negative for mycoplasma).

### Isolation of leukocytes from solid human tissues and peripheral blood

After surgical procedures, fresh tissue samples were placed immediately into a 50-ml conical tube with complete media (RP10: RPMI1640 supplemented with penicillin, streptomcyin and 10% fetal bovine serum (FBS)) and kept at 4 °C. Samples were processed within 1–4 h after collection based on optimized protocols adapted from ref. ^53^. In brief, tissue pieces were minced using a scalpel into small pieces and incubated with Collagenase II (Sigma-Aldrich, 0.7 mg ml^−1^) and DNAse (5 U ml^−1^) in RPMI1640 with 7.5% FBS for 30–45 min depending on sample size. Subsequently, any remaining tissue pieces were mechanically disrupted by repeated resuspension with a 30 ml syringe with a large bore tip (16 × 1.5 blunt). The cell suspension was filtered using a 70-μm cell strainer, washed in RPMI1640 and immediately used for downstream procedures.

Peripheral blood samples (1–10 ml) were collected in ACD tubes and then processed using SepMate tubes (StemCell Technologies, 85450) and Lymphoprep (Stem Cell Technologies, 07851) according to manufacturer protocols. In brief, whole blood samples were centrifuged for 10 min at 400*g*, and the plasma supernatant was collected separately and immediately frozen at −80 °C. Remaining cells were resuspended in 30 ml PBS and pipetted on top of 13.5 ml Lymphoprep in a SepMate tube. After centrifugation for 16 min at 1,200*g*, the mononuclear cell fraction in the supernatant was poured into a fresh 50-ml tube, washed with PBS and immediately used for downstream procedures. For blood samples from dental surgery patients, red blood cells were lysed using ACK-lysis buffer (Thermo Fisher, A10492-01), and the remaining white blood cells were directly used for downstream staining.

If required, cells isolated from tissue samples or from peripheral blood were frozen using either a 90% FBS/10% DMSO mixture or Cell Culture Freezing Medium (Gibco, 12648010), and stored in liquid nitrogen until used for downstream procedures.

### Flow cytometry and cell sorting

For flow cytometric analysis good practices were followed as outlined in the guidelines for use of flow cytometry^54^ and consensus suggestions for data analysis^55^. Directly following isolation or thawing, cells were incubated with Fc-blocking reagent (BioLegend Trustain FcX, 422302) and fixable UV Blue Live/Dead reagent (ThermoFisher, L34961) in PBS (Gibco, 14190250) for 15 min at room temperature. After this, cells were incubated for 20 min at room temperature with 50 μl total volume of antibody master mix freshly prepared in Brilliant staining buffer (BD Biosciences, 563794), followed by two washes in fluorescence-activated cell sorting (FACS) buffer (PBS with 2% FBS). All antibodies were titrated and used at optimal dilution, and staining procedures were performed in 96-well round-bottom plates (for cell sorting in 5-ml polystyrene tubes). A detailed list of the main panels used, including fluorochromes, antibody catalogue numbers and final dilutions is provided in Supplementary Table 2 (panels designed according to best practices as described^56^) and Supplementary Table 3. For sorting, cells were immediately used after staining, and for analysis, the stained cells were fixed with 4% PFA (Cytofix/Cytoperm, BD Biosciences, 554722) for 20 min at room temperature, washed, resuspended in FACS buffer and stored at 4 °C in the dark until acquisition. If necessary, intracellular (CD68, granzyme B (GZMB) or CTLA4) or intranuclear staining (FOXP3, KI67, TCF1, TOX, T-bet or EOMES) was performed following the appropriate manufacturer protocols (eBioscience FOXP3/Transcription Factor Staining Buffer Set, Thermo Fisher 00-5532-00).

Single-stained controls were prepared with every experiment using antibody capture beads (BD Biosciences anti-mouse (552843) or anti-mouse Plus, and anti-rat (552844)) diluted in FACS buffer, or cells for Live/Dead reagent, and treated exactly the same as the samples (including fixation procedures). For each staining of experimental samples, a PBMC sample from the same healthy donor (SAC) was stained with the same panel as a longitudinal reference control (data not shown).

All samples were acquired using a FACSymphony A5 (BD Biosciences), equipped with 30 detectors and 355 nm (65 mW), 405 nm (200 mW), 488 nm (200 mW), 532 nm (200 mW) and 628 nm (200 mW) lasers and FACSDiva acquisition software (BD Biosciences). Full details on the optical configuration of the instruments used are as described^19^. Detector voltages were optimized using a modified voltage titration approach^57^ and standardized from day to day using MFI target values and 6-peak Ultra Rainbow Beads^56^ (Spherotec, URCP-38-2K). After acquisition, data was exported in FCS 3.1 format and analysed using FlowJo (version 10.6.x, and 10.7.x, BD Biosciences). Samples were analysed using a combination of manual gating and computational analyses approaches^55^, with doublets being excluded by FSC-A vs FSC-H gating. For fresh samples acquired on different experimental days with the T cell or APC panel, files were exported as compensated data and analysed combined together in a new workspace (see deposited data on www.flowrepository.org). Gates were kept the same across all samples except where changes in the density distribution of populations clearly indicated the need for sample-specific adjustment. For the APC panel, PD-L1 (V450 channel) as well as CD85k (V510 channel) were excluded from analysis because of interference or high variability from highly auto-fluorescent myeloid cells in some samples. For the T cell panel, granzyme B and TIM3 staining showed donor-specific shifts in intensity, requiring sample-specific adjustments of gates.

All cell sorting was performed either on a FACSAria III (BD Biosciences), equipped with 20 detectors and 405 nm, 488 nm, 532 nm and 628 nm lasers or on a FACSymphony S6 cell sorter (BD Biosciences), equipped with 50 detectors and 355 nm, 405 nm, 488 nm, 532 nm and 628 nm lasers. For all sorts involving myeloid cells, an 85-μm nozzle operated at 45 psi sheath pressure was used, for sorts exclusively targeting T cells, a 70-μm nozzle at 70 psi sheath pressure was used. Unless stated otherwise, cells were sorted into chilled Eppendorf tubes containing 500–1,000 μl complete RPMI, washed once in PBS and immediately used for subsequent processing.

### Whole-transcriptome single-cell library preparation and sequencing

cDNA libraries were generated using the 10x Genomics Chromium Single Cell 3′ Reagent Kits v2 protocol or the v3 protocol, or using the 10x Genomics Chromium Single Cell 5′ Reagent Kit v1 protocol (see Supplementary Table 1). In brief, after sorting single cells were isolated into oil emulsion droplets with barcoded gel beads and reverse transcriptase mix using the Chromium controller (10x Genomics). cDNA was generated within these droplets, then the droplets were dissociated. cDNA was purified using DynaBeads MyOne Silane magnetic beads (ThermoFisher, 370002D). cDNA amplification was performed by PCR (10 cycles) using reagents within the Chromium Single Cell 3′ Reagent Kit v2 or v3 (10x Genomics) or the VDJ and GEX reagent kit v1 (see list of samples in Supplementary Table 1). Amplified cDNA was purified using SPRIselect magnetic beads (Beckman Coulter) according to the respective protocol. cDNA was enzymatically fragmented and size selected prior to library construction. Libraries were constructed by performing end repair, A-tailing, adaptor ligation, and PCR (12 cycles). Quality of the libraries was assessed by using Agilent 2200 TapeStation with High Sensitivity D5000 ScreenTape (Agilent). Quantity of libraries was assessed by performing digital droplet PCR (ddPCR) with Library Quantification Kit for Illumina TruSeq (BioRad, 1863040) or determined by Qubit with the dsDNA HS Assay (Q32851). Pooled Libraries were diluted to 2 nM or 3 nM and paired-end sequencing was performed on a HiSeq 2500 (Illumina) or a NovaSeq 6000 (Illumina) utilizing S1 or S2 flow cells, targeting between 25,000–50,000 reads per cell.

### Targeted transcriptomics single-cell library preparation and sequencing

cDNA libraries were generated as described in detail^58^. In brief, after sorting, single cells were stained with Sample-Tag antibodies (if required, see Extended Data Fig. 8a) and or AbSeq antibodies (if required), washed three times, pooled and counted and subsequently loaded onto a nano-well cartridge (BD Rhapsody), lysed inside the wells followed by mRNA capture on cell capture beads according to manufacturer instructions^58^. Cell Capture Beads were retrieved and washed prior to performing reverse transcription and treatment with Exonuclease I. cDNA underwent targeted amplification using the Human Immune Response Panel primers and a custom supplemental panel (listed in Supplementary Table 3) via PCR (10–11 cycles). PCR products were purified, and mRNA PCR products were separated from Sample-Tag (and AbSeq, where applicable) PCR products with double-sided size selection using SPRIselect magnetic beads (Beckman Coulter). mRNA and Sample Tag products were further amplified using PCR (ten cycles). PCR products were then purified using SPRIselect magnetic beads. Quality of PCR products was determined by using an Agilent 2200 TapeStation with High Sensitivity D5000 ScreenTape (Agilent) in the Fred Hutch Genomics Shared Resource laboratory. The quantity of PCR products was determined by Qubit with Qubit dsDNA HS Assay (Q32851). Targeted mRNA product was diluted to 2.5 ng μl^−1^, and the Sample Tag and AbSeq PCR products were diluted to 1 ng μl^−1^ to prepare final libraries. Final libraries were indexed using PCR (6 cycles). Index PCR products were purified using SPRIselect magnetic beads. Quality of all final libraries was assessed by using Agilent 2200 TapeStation with High Sensitivity D5000 ScreenTape and quantified using a Qubit Fluorometer using the Qubit dsDNA HS Kit (ThermoFisher). Final libraries were diluted to 3 nM and multiplexed for paired-end (100 bp) sequencing on a NovaSeq 6000 (Illumina) using S1 and S2 flow cells. For the gene expression library, we targeted 5,000–20,000 reads per cell, for the AbSeq library 10,000–15,000 reads per cell, and for the Sample-Tag libraries 500–2,000 reads per cell.

### Ex vivo stimulation assays

Cells were isolated from tissues or blood as described above. For some of the stimulation assays cryo-preserved cell suspensions were used after assessing good cellular viability. For the 2 h short-term stimulation assays with targeted transcriptomics (Fig. 4), CD3^+^ T cells (live CD45^+^CD19^−^CD3^+^ events) were isolated using FACS using a BD FACSAria II. Five-thousand cells were placed into each well of a V-bottom 96-well plate with 200 μl complete media. Cells were then left untreated (control), or stimulated with IL-12, IL-15 and IL-18 (each at 1 nM), or with PMA (50 ng ml^−1^) and ionomycin (500 ng ml^−1^) for 2 h at 37 °C. Cells were then washed with 1× PBS and prepared for targeted transcriptomics and staining with oligonucleotide-conjugated antibodies as described^58^. For the 1- to 3-day stimulation assays (Fig. 4, Extended Data Fig. 7), CD4^+^CD25^+^CD127^−^IL1R1^+^ and IL1R1^−^ T_reg_ cells were isolated from blood and HNSCC tissues using a FACSymphony S6 sorter (BD Biosciences), and cultured either in RP10 alone or with anti-CD3/CD28 Dynabeads (Gibco, 11161D, used at a 1:1 bead-to-cell ratio) or with anti-CD3/CD28/CD2 beads (Miltenyi, 130-092-909, T_reg_ Suppression Inspector, also used at a 1:1 bead-to-cell ratio), either with or without recombinant IL-1β (Peprotech, 200-01B) at 50 ng ml^−1^. For some experiments, culture cells were subsequently stained and 250–500 viable cells were sorted on an BD S6 sorter followed by bulk RNA-sequencing (RNA-seq) analysis using the SMART-Seq v4 kit (Takara) as described further below.

### Suppression assays

For suppression assays, IL1R1^+^ and IL1R1^−^CD4^+^CD25^+^CD127^−^ regulatory T cells and CD4^+^CD25^−^ and CD8^+^ T_resp_ cells were sorted from cryopreserved HNSCC samples. For some experiments, matched peripheral blood was included. T_resp_ cells were labelled with Cell Trace Violet (CTV) according to the manufacturer instructions (Thermo Fisher, C34571). In brief, 10^6^ sorted T_resp_ cells were washed with PBS after the sort, and then incubated in pre-warmed PBS containing a final concentration of 5 µM freshly diluted CTV for 15 min. The reaction was quenched with prewarmed RP10. Both T_resp_ and T_reg_ cells were counted twice on a BioRad TC20 cell counter. 10,000 (20,000 for some experiments) CTV-labelled T_resp_ cells were cultured alone, or with 10,000 T_reg_ cells (or titred amounts of T_reg_ cells) in a 96-well round-bottom plate at 37 °C for 4 days together with anti-CD3/CD28/CD2 beads (Miltenyi, 130-092-909, T_reg_ Suppression Inspector). An unstimulated control well was included with every experiment. Where indicated, recombinant IL-1β (Peprotech, 200-01B) was added to achieve a final concentration of 50 ng ml^−1^. On the read-out day, supernatants were collected and frozen at −80 °C, and the cells were stained with a 14-colour readout panel including Live/Dead reagent (Supplementary Table 2), fixed and acquired on a BD FACSymphony A5, as described above. Cell proliferation was assessed by using the proliferation platform in FlowJo 10.7 (BD Biosciences), with percentage of divided cells (modelled, not gated) as the main readout. Supernatants were processed for Luminex analysis by the Immunomonitoring Core of the Fred Hutchinson Cancer Research Center.

### Luminex analysis of tumour lysates

Luminex analysis was performed on lysates of tissues. To obtain lysates from tumour tissues, a 2 × 2 mm piece was incubated for one minute in PBS/0.1% tween. After incubation, the tissue piece was minced in the buffer and then centrifuged at 10,000 rpm for 5 min. The supernatant was collected and immediately flash-frozen on dry ice. Processing for Luminex was performed by the Immunomonitoring Core of the Fred Hutchinson Cancer Research Center.

### Isolation and stimulation of mouse cells

Mouse protocols were approved by and in compliance with the ethical regulations of Fred Hutchinson Cancer Research Center’s IACUC. All animals were maintained in specific pathogen-free facilities and euthanized in accordance with institutional protocols. We received thymus, spleen, and lymph node (LN) from male *Foxp3*^*eGFP-cre-ERT2*^ mice (age ≥8 weeks) (from J. Lund), and mechanically dissociated thymus, spleen or lymph node through a 70-µm strainer. To enrich T cells from spleen–lymph node single-cell suspensions, we used a T cell-negative isolation based magnetic enrichment (Stemcell Technologies). For TCR stimulations, we prepared plate-bound anti-CD3 and anti-CD28 by incubating 96-well V-bottom tissue culture plates with 100 µl of 1 µg ml^−1^ anti-CD3 (clone: 145-2C11) and 2 µg ml^−1^ anti-CD28 (clone 37.51) in 1× PBS for 3 h at 37 °C. We decanted and washed residual anti-CD3/anti-CD28 solution and plated 1 × 10^6^ isolated T cells per well in 96-well V-bottom tissue culture plates. We cultured cells in modified RP10 media (RPMI1640 supplemented with 10% FBS, 2mM l-glutamine, 100 U ml^−1^ penicillin-streptomycin, 1 mM sodium pyruvate, 0.05 mM β-mercaptoethanol and 1 mM HEPES). We collected cells for flow analysis at 0-, 1- and 2-day time points for flow cytometric analysis as described above. The following panel was used: anti-TCRγδ–PerCPe710 (clone eBioGL3), anti-CD4–BV786 (clone GK1.5), anti-CD8a–V500 (clone 53-6.7), anti-CD44–AF700 (clone IM7), anti-CD69–PECy7 (clone H1.2F3), anti-PD-1–BV605 (clone 28F.1A12), anti-ICOS–AF647 (clone C398.4A), anti-IL1R1–PE (clone 35F5), anti-IL1R2–BV421 (clone 4E2), anti-CD3–BUV805 (clone 17A2) and anti-FOXP3–FITC (clone FJK-16s, intranuclear post fixation).

### Humanized mouse experiments

MISTRG mice (M-CSF^h/h^IL-3/GM-CSF^h/h^SIRPα^h/m^TPO^h/h^RAG2^−/−^IL2Rγ^−/−^) were previously reported^59^. All animal experiments were approved by Fred Hutchinson Cancer Research Center’s Institutional Animal Care and Use Committee (protocol 50941). De-identified human fetal liver tissues, obtained with informed consent from the donors, were procured by Advanced Bioscience Resources and their use was determined as non-human subject research by Fred Hutch’s Institutional Review Board (6007-827). Fetal livers were cut in small fragments, treated for 45 min at 37 °C with collagenase D (Roche, 100 ng ml^−1^), and a single-cell suspension was prepared. Hematopoietic cells were enriched by density gradient centrifugation in Lymphocyte Separation Medium (MP Biomedicals) followed by positive immunomagnetic selection with anti-human CD34 microbeads (Miltenyi Biotec). Purity (>90% CD34^+^ cells) was confirmed by flow cytometry and cells were frozen at −80 °C in FBS containing 10% DMSO. Newborn MISTRG mice (day 1–3) were sublethally irradiated (80 cGy gamma rays in a Caesium-137 irradiator) and ∼20,000 CD34^+^ cells in 20 μl PBS were injected into the liver with a 22-gauge needle (Hamilton Company), as described^59^. Engraftment levels were measured as the percentage of human CD45^+^ cells among total (mouse and human combined) CD45^+^ cells in the blood.

The human squamous cell carcinoma line SCC-15 was obtained and verified from ATCC. Cells were grown to ∼80% confluency in DMEM/F12 supplemented with 12.5 mM l-glutamine, 15 mM HEPES, 0.5 mM sodium pyruvate and 400 ng ml^−1^ hydrocortisone. Approximately 0.5 million cells per mouse were resuspended in 75 µl PBS, mixed with 25 µl growth-factor-reduced Matrigel (Corning, 354230) and then injected subcutaneously under anaesthesia in the flank of humanized mice. The size of the tumours was measured weekly for 7 weeks with a calliper. SCC15 tumour tissues were processed for leukocyte isolation as described above for human tissues.

### Bulk RNA-seq experiments and analysis

Bulk RNA-seq was performed on 250 sort-purified IL1R1^+^ and IL1-R1^−^ T_reg_ cells derived from either cryopreserved blood or HNSCC tissues samples after culture in conditions of no stimulation, stimulation with anti-CD3/CD28/CD2 beads, and stimulation with anti-CD3/CD28/CD2 beads and IL-1β (50 ng ml^−1^) for days 1, 2, and 3. In total, 88 samples were sequenced, and each condition was represented by at least 3 or more biological replicates.

Cells were sorted directly into lysis buffer from the SMART-Seq v4 Ultra Low Input RNA Kit for sequencing (Takara), immediately snap frozen on dry ice, and transferred to −80 °C storage until processed into cDNA. All samples were thawed, cells were lysed, and cDNA was synthesized and amplified per the manufacture’s instruction. After amplification, sequencing libraries were constructed using the NexteraXT DNA sample preparation kit with unique dual indexes (Illumina) to generate Illumina-compatible barcoded libraries. Libraries were pooled and quantified using a Qubit Fluorometer (Life Technologies). Sequencing of pooled libraries was carried out on a NextSeq 2000 sequencer (Illumina) with paired-end 59-base reads, using a NextSeq P2 sequencing kit (Illumina) with a target depth of 5 million reads per sample.

Base calls were processed to FASTQs on BaseSpace (Illumina), and a base call quality-trimming step was applied to remove low-confidence base calls from the ends of reads. Reads were processed using workflows managed on the Galaxy platform. Reads were trimmed by 1 base at the 3′ end then trimmed from both ends until base calls had a minimum quality score of at least 30. Any remaining adapter sequence was removed as well. To align the trimmed reads, STAR aligner (v2.4.2a) was used with the GRCh38 reference genome and gene annotations from ensembl release 91. Gene counts were generated using HTSeq-count (v0.4.1). Quality metrics were compiled from PICARD (v1.134), FASTQC (v0.11.3), Samtools (v1.2), and HTSeq-count (v0.4.1).

A quality filter was applied to retain libraries in which the fraction of aligned reads examined compared to total FASTQ reads was >70%, the median coefficient of variation of coverage was less than 0.85, and the library had at least 1 million reads. All sequenced samples passed these quality filters. Non-protein coding genes and genes expressed at less than 1 count per million in fewer than 10% of samples were filtered out. Expression counts were normalized using the TMM algorithm. For differential gene expression analysis, the linear models for microarray data (Limma) R package after Voom transformation was used; this approach either outperforms or is highly concordant with other published methods. Linear models were generated, and donor identity was included as a random effect. For differential gene expression comparisons, genes with a false discovery rate (FDR) of less than 0.1 and an absolute expression fold-change greater than 1 were considered differentially expressed.

### Pre-processing for whole transcriptome analysis (WTA) and targeted transcriptomics data

Raw base call (BCL) files were demultiplexed to generate Fastq files using the Cell Ranger mkfastq pipeline within Cell Ranger (10x Genomics). Whole-transcriptome Fastq files were processed using the standard Cell Ranger pipeline (10x genomics) within Cell Ranger 2.1.1 or Cell Ranger 3.0.2. In brief, Cell Ranger count performs read alignment, filtering, barcode and unique molecular identifier (UMI) counting, and determination of putative cells. The final output of Cell Ranger (the molecule per cell count matrix) was then analysed in R using the package Seurat^60,61^ (3.0) as described below. For targeted transcriptomics data, Fastq files were processed via the standard Rhapsody analysis pipeline (BD Biosciences) on Seven Bridges (www.sevenbridges.com). In brief, after read filtering, reads are aligned to a reference genome and annotated, barcodes and UMIs are counted, followed by determining putative cells. The final output (molecule per cell count matrix) was also analysed in R using Seurat^60,61^ (version 3.0) as described below. For 5′ VDJ sequencing experiments, the output after Cell Ranger vdj was analysed using the Loupe VDJ browser v3 (10x Genomics). For the SMART-Seq v4 experiments, Fastq files were aligned to the GRCh38 reference genome as described in more detail above.

### Seurat workflow for targeted transcriptomics and WTA data

The R package Seurat^60,61^ was used for all downstream analysis, with custom scripts based on the following general guidelines for analysis of scRNA-seq data^62^.

In brief, for whole-transcriptome data, only cells that had at least 200 genes (v2 kits) or 800 genes (v3 kits), and depending on sample distribution less than 7–15% mitochondrial genes were included in analysis. All acquired samples were merged into a single Seurat object, followed by a natural log normalization using a scale factor of 10,000, determination of variable genes using the vst method, and a *z*-score scaling. Principal component analysis was used to generate 75 principal components, followed by data integration using Harmony^30^. The dimensionality reduction generated by Harmony was used to calculate UMAP, and graph-based clustering with a resolution between 0.2 and 0.6. For cell annotation, we applied SingleR as a purely data-driven approach^32^, and used the expression of typical lineage transcripts to verify the cell label annotation. For all subsequent analysis steps, the integrated Seurat object was separated into two objects containing all T cells or all APCs, respectively, and UMAP calculation as well as clustering steps were repeated.

For targeted transcriptomics data^36^, separate cartridges from the same experiment were merged (if applicable), and only cells that had at least 30 genes were included in downstream analysis. After generating a Seurat object, a natural log normalization using a scale factor of 10,000 was done, followed by determination of variable genes using the vst method, and a *z*-score scaling. Principal component analysis was used to generate 75 principal components, followed by data integration using Harmony^30^. The dimensionality reduction from Harmony was used for subsequent UMAP calculation and graph-based clustering with tuned resolution. Protein phenotyping data was stored in a separate slot as described in the Seurat tutorial for CITE-seq data, and normalized using the centred log ratio (CLR) method^36^. For some figures, the count matrices were exported as FCS files using the package Premessa, and then imported into FlowJo 10.7.x. Appropriate arcsinh transformations were applied in a channel-specific manner, and transcript or protein expression was plotted and quantified using two-dimensional plots.

For all differential gene expression analyses we utilized the Seurat implementation of MAST (model-based analysis of single-cell transcriptomes) with the number of UMIs included as a covariate (proxy for cellular detection rate (CDR)) in the model^34^. For calculating the T helper scores (Extended Data Fig. 5f, g) we used the AddModuleScore function of Seurat (see Github script on https://github.com/MairFlo/Tumor_vs_Inflamed/blob/main/OM_HNSCC_scRNAseq_Harmony). The genes used were as follows: T_H_1: *IFNG*, *TBX21*, *IL12RB1* and *IL12RB2*; T_H_2: *TNFSF11*, *GATA3* and *IL4*; T_H_17: *RORC*, *CCR6*, *IL17A*, *IL17F*, *IL23R*, *IL22*, *AHR*, *IL26*, *CCL20*; T_C_: *CD8B*, *CD8A*, *TNF*, *IFNG*, *IL2*, *GZMB*, *PRF1*, *GZMA* and *FAS*. T_ex_: *TCF7*, *TOX*, *HAVCR2*, *PDCD1* and *LAG3*; T_reg_: *FOXP3*, *CTLA4*, *IL2RA*, *IL2RB* and *ENTPD1*.

### NicheNet workflow

NicheNet analysis was adapted from the vignette described at https://github.com/saeyslab/nichenetr^35^. In brief, the separate Seurat objects containing APCs (described above) were subsetted to contain only HNSCC-derived cells, and the Seurat object containing T cells only HNSCC and OM-derived cells. During multiple separate NicheNet runs, different T cell subsets were set as ‘receiver’ (that is, CD4 non-T_reg_ clusters 0 and 2, CD8 T cell clusters 1, 3 and 4 and T_reg_ cluster 5; Extended Data Fig. 5a) and all myeloid cell clusters (except the pDC and mast cell cluster; Fig 2b) as ‘sender’ populations. For the receiver cell population, a DEG test was performed to find genes enriched in HNSCC vs OM samples, with the key parameters being set as follows: genes expressed in at least 10% of the cells of the respective T cell clusters, and filtered after the DEG test for an adjusted *P*-value of less than 0.05 and average log fold change more than 0.25. For the sending cell population, all ligands expressed in at least 5% of the cells in the respective APC cluster were considered. NicheNet analysis was performed based on the vignette to infer receptors, filter for documented links and generate a circus plot of the top ligand-receptor interactions for the respective cellular populations. Scoring of the predicted targets was based on a Pearson correlation coefficient as described in the NicheNet vignette. Circos plots were generated as described in the vignette^35^ to visualize links between ligands on APCs and receptors on the T cell subsets.

### FAUST analysis

For the T cell panel, FAUST was used to discover and annotate phenotypes in 22 samples (11 HNSCC and 11 OM). FAUST was applied to CD45^+^ live lymphocytes identified through manual gating. The MR1–tetramer, CD45 and the Live/Dead marker were excluded from the FAUST analysis to account for the manual analysis. After tuning, FAUST selected the markers CD8, CD4, CD3, CD45RA, CD27, CD19, CD103, CD69, CD28, HLADR, GZMB, PD-1, CD25, ICOS, TCRγδ, CD38 and TIM3 for discovery and annotation of phenotypes. Counts of the discovered phenotypes labelled CD3^+^ and CD19^−^ were tested for association with tissue type using a binomial generalized linear mixed-effects model with a subject level random effect. Fifty phenotypes were associated with tissue type at the FDR-adjusted 0.05 level.

For the APC panel, FAUST was used to discover and annotate phenotypes in 32 samples (16 HNSCC and 16 OM). FAUST was applied to CD45^+^ live CD19^−^CD3^−^ cells identified through manual gating. The markers CD3, CD19, CD45, PD-L2 and CD85k and the Live/Dead marker were excluded from the FAUST analysis to account for the manual analysis as well as observed autofluorescence in the detectors used for PD-L2 and CD85k. After tuning, FAUST selected the markers CD1c, CD11b, CD11c, CD14, CD16, CD32, CD38, CD40, CD68, CD80, CD86, CD123, CD141, CD163, CD206, CX3CR1, HLADR, PDL1 and SIRPA for discovery and annotation of phenotypes. Counts of the discovered phenotypes annotated as HLADR^+^ were tested for association with tissue type using a binomial generalized linear mixed-effects model with a subject level random effect. 21 phenotypes were associated with tissue type at the FDR-adjusted 0.05 level.

### Statistical analyses

Unless stated otherwise, all data are represented as mean ± s.d. Statistical analyses between blood, OM and HNSCC samples were performed using one-way ANOVA with Tukey’s multiple comparisons test. *P*-values are shown in full, except if smaller than 0.0001. Statistical analysis was performed using GraphPad Prism (v9).

### Reporting summary

Further information on research design is available in the Nature Research Reporting Summary linked to this paper.

## Online content

Any methods, additional references, Nature Research reporting summaries, source data, extended data, supplementary information, acknowledgements, peer review information; details of author contributions and competing interests; and statements of data and code availability are available at 10.1038/s41586-022-04718-w.

## Supplementary information

Supplementary Information This file contains Supplementary Tables 2–4 and Figs. 1, 2.

Reporting Summary

## Data Availability

The scRNA-seq data as well as the bulk RNA-seq data discussed in this publication have been deposited in the NCBI Gene Expression Omnibus under accession GSE163633. Alignment was based on the GRCh38 reference genome. Flow cytometry raw data have been deposited at https://www.flowrepository.org using the Identifiers FR-FCM-Z4UX, FR-FCM-Z4UP and FR-FCM-Z4UQ or can be requested from F.M (fmair@fredhutch.org). The data used in Extended Data Fig. 9c–e are from the scRNA-seq Data Portal for T cells in Pan Cancer at http://cancer-pku.cn:3838/PanC_T.

## Code availability

The main R scripts used for data processing of the scRNA-seq data are available on https://github.com/MairFlo/Tumor_vs_Inflamed or can be requested from F.M. The scripts used for processing of the bulk RNA-seq data are available on https://github.com/akonecny/Bulk-RNAseq-Tumor_vs_Inflamed.
