# Supplementary material for: Extricating human tumour immune alterations from tissue inflammation
Source: Nature. 2022 May 11;605(7911):728–35. doi: 10.1038/s41586-022-04718-w (PMC9132772; doi:10.1038/s41586-022-04718-w)
Supplement: Supplementary file 1 — This file contains Supplementary Tables 2–4 and Figs. 1, 2. [file 41586_2022_4718_MOESM1_ESM.pdf]

nature portfolio

https://doi.org/10.1038/s41586-022-04718-w

Supplementary information

Extricating human tumour immune alterations from tissue inflammation

In the format provided by the authors and unedited

Extricating human tumor-enriched immune alterations from non-malignant tissue inflammation

Florian Mair[#1], Jami R. Erickson[#1,8], Marie Frutoso[1], Andrew J. Konecny[1,8], Evan Greene[1], Valentin Voillet[1,5], Nicholas J. Maurice[1], Anthony Rongvaux[3,8], Douglas Dixon[2§], Brittany Barber[4], Raphael Gottardo[1,6**] and Martin Prlic[1,7*]

[#]These authors contributed equally

* Correspondence: mprlic@fredhutch.org

Supplementary information table of contents:

Supplementary Table 1: List of tissue types analyzed

Supplementary Table 2: Flow cytometry panels used

Supplementary Table 3: List of antibodies used

Supplementary Table 4: Custom gene panel targeted transcriptomics

Supplementary Table 1
List of tissue types analyzed.
10x = 10x genomics scRNA-seq experiment
Rhaps = Rhapsody (targeted transcriptomics and/or AbSeq)
Suppr. assay = T cell/Treg suppression assay

Type	ID	Tissue source	Infl Score	APC panel	T cell panel	IL1R1 panel	Sort and *In vitro* assay	sc-RNAseq
OM	116	dental: sulcular and attached gingiva	High	Yes				
OM	119	dental: sulcular and attached gingiva	Low	Yes	Yes			
OM	121	implant: sulcular and attached gingiva	High					10x v2
OM	122	dental: sulcular and attached gingiva	Low					10x v2
OM	124	dental: sulcular and attached gingiva	High					10x v2
OM	125	dental: sulcular and attached gingiva	High					10x v2
OM	127	dental: sulcular and attached gingiva	High	Yes	Yes			
OM	128	dental: sulcular and attached gingiva	High	Yes	Yes			
OM	136	dental: sulcular and attached gingiva	High	Yes				
OM	138	dental: sulcular and attached gingiva	Low	Yes	Yes			
OM	139	dental: sulcular and attached gingiva	Low	Yes	Yes			
OM	141	dental: sulcular and attached gingiva	Low	Yes	Yes			
OM	145	dental: sulcular and attached gingiva	Low	Yes				
OM	152	dental: sulcular and attached gingiva	NA	Yes	Yes			
OM	153	implant: sulcular and attached gingiva	Low	Yes	Yes			
OM	161	dental: sulcular and attached gingiva	Low		Yes			
OM	162	dental: sulcular and attached gingiva	NA	Yes	Yes			
OM	163	dental: sulcular and attached gingiva	NA	Yes	Yes			
OM	167	implant: sulcular and attached gingiva	High	Yes				
OM	169	dental: sulcular and attached gingiva	Low	Yes	Yes			
OM	170	implant: sulcular and attached gingiva	Low	Yes				
OM	183	dental: osseous keratinized gingiva	NA			Yes		
OM	184	dental: sulcular keratinized gingiva	NA			Yes		
OM	190	dental: sulcular keratinized gingiva	NA			Yes		
OM	191	dental: NA	NA			Yes		
OM	192	dental: NA	NA			Yes		
OM	193	dental: sulcular keratinized gingiva	NA			Yes		

Type	ID	Tissue source	Infl Score	APC panel	T cell panel	IL1R1 panel		sc-RNAseq
HNSCC	2	base of tongue SCC	NA					10x v2
HNSCC	4	base of tongue SCC	NA	Yes	Yes		TCR stim	Rhaps and Bulk RNAseq
HNSCC	5	mandible SCC	NA				TCR stim	Bulk RNAseq
HNSCC	6	oral tongue SCC	NA	Yes	Yes			
HNSCC	7	oral tongue SCC	NA	Yes	Yes			Rhaps
HNSCC	7b	tonsil SCC	NA	Yes	Yes			
HNSCC	8	oral tongue SCC	NA	Yes	Yes			Rhaps
HNSCC	11	tonsil SCC	NA	Yes	Yes			
HNSCC	12	tonsil SCC	NA	Yes	Yes			
HNSCC	13	oral tongue SCC	NA					Rhaps+AbSeq
HNSCC	14	laryngeal SCC	NA	Yes	Yes			Rhaps+AbSeq
HNSCC	15	oral tongue SCC	NA	Yes	Yes			10x v2
HNSCC	17	tonsil SCC	NA	Yes			TCR stim	Bulk RNAseq
HNSCC	18	metastatic SCC to cervical lymph node	NA	Yes	Yes			Rhaps+AbSeq
HNSCC	19	base of tongue SCC	NA	Yes	Yes			
HNSCC	20	base of tongue SCC	NA					10x v3
HNSCC	21	maxillary sinus SCC	NA	Yes				
HNSCC	22	metastatic SCC to cervical lymph node	NA		Yes			
HNSCC	23	tonsil SCC	NA			Yes		
HNSCC	27	laryngeal SCC	NA	Yes	Yes			10x v3
HNSCC	28	palatal lesion SCC	NA			Yes		
HNSCC	29	floor of mouth SCC	NA			Yes		
HNSCC	30	oral tongue SCC	NA	Yes	Yes			
HNSCC	35	base of tongue SCC	NA	Yes				
HNSCC	48	oral tongue SCC	NA	No	No	Yes		
HNSCC	49	base of tongue	NA				Suppr assay	Rhaps+AbSeq
HNSCC	51	oral tongue SCC	NA					Rhaps+AbSeq
HNSCC	52	pharyngeal SCC	NA				Suppr assay	
HNSCC	53	base of tongue SCC	NA			Yes		10x VDJ v1
HNSCC	56	tonsil SCC	NA				Suppr assay	
HNSCC	68	mandible SCC	NA				Suppr assay	
HNSCC	69	base of tongue SCC	NA					10x VDJ v1
HNSCC	72	pharyngeal SCC	NA				Suppr assay	

HNSCC	73	glossotonsillar SCC	NA		Suppr assay	
HNSCC	75	pharyngeal SCC	NA		Suppr assay	
HNSCC	77	oral tongue SCC	NA			10x VDJ v1
HNSCC	79	tongue SCC	NA		Suppr assay	
HNSCC	84	neck mass SCC	NA	Yes		
HNSCC	86	tongue SCC	NA		Suppr assay	
HNSCC	89	mandible SCC	NA		Suppr Assay	
HNSCC	90	laryngeal SCC	NA	Yes		
HNSCC	93	sinonasal SCC	NA	Yes	TCR stim	Bulk RNAseq
HNSCC	91	tonsil SCC	NA		Suppr assay	
HNSCC	98	glossotonsillar SCC	NA		Suppr assay	Bulk RNAseq
HNSCC	99	tongue SCC	NA		TCR stim	Bulk RNAseq
HNSCC	101	base of tongue SCC	NA		TCR stim	Bulk RNAseq
HNSCC	102	tonsil SCC	NA		Suppr assay	Bulk RNAseq
HNSCC	103	floor of mouth SCC	NA		TCR stim	Bulk RNAseq
Other	54	Papillary carcinoma	NA	Yes		
Other	61	Papillary carcinoma	NA	Yes		
Other	64	Papillary carcinoma	NA	Yes		Rhaps+AbSeq
Other	65	Papillary carcinoma	NA	Yes		
Other	76	Breast Cancer	NA	Yes		
Other	47	Lung Cancer	NA	Yes		

Supplementary Table 2

High-dimensional flow cytometry panels used

For panel development details, see Mair et al, Cytometry Part A 2018

ic: stained intracellularly after fixation+permeabilization

PANEL 1: APC panel

		Fluorophore	Antigen	Dilutions	
1	355nm	BUV395	CD40	1: 40	
2		L/D UV Blue	L/D	1: 500	
3		BUV496	CD16	1: 320	
4		BUV563	CD56	1: 160	
5		BUV661	CD3	1: 80	
6		BUV737	CD86	1: 40	
7		BUV805	CD45	1: 80	
8	405nm	BV421	PD-L2	1: 20	
9		BV480	CD85k	1: 40	
10		BV570	CD14	1: 20	
11		BV605	CD141	1: 640	
12		BV650	Sirpa	1: 160	
13		BV711	*CD68*	*1: 40*	*ic*
14		BV750	CD11b	1: 160	
15		BV785	CD123	1: 40	
16	488nm	BB515	CD206	1: 20	
17		BB630	BTLA	1: 80	
18		BB660	PD-L1	1: 40	
19		BB700	CD32	1: 160	
20		BB790	CD38	1: 80	
21	532nm	PE	Axl	1: 20	
22		PE-CF594	CD163	1: 40	
23		PE-Cy5	CD80	1: 10	
24		PE-Cy5.5	CD19	1: 160	
25		PE-Cy7	CX3CR1	1: 160	
26	628nm	AF647	CD1c	1: 160	
27		AF700	CD11c	1: 320	
28		APC-H7	HLA-DR	1: 40	

PANEL 2: general T cell panel

		Fluorophore	Antigen	Dilutions
1	355nm	BUV395	CD8	1: 80
2		L/D UV Blue	L/D	1: 500
3		BUV496	CD3	1: 40
4		BUV563	CD25	1: 40
5		BUV661	HLA-DR	1: 80
6		BUV737	ICOS	1: 10
7		BUV805	CD45	1: 80
8	405nm	BV421	MR1Tet	1: 500
9		BV480	CD28	1: 40
10		BV570	CD45RA	1: 160
11		BV605	PD1	1: 20
12		BV650	CD69	1: 20
13		BV711	OX40	1: 40
14		BV750	CD103	1: 160
15		BV785	IL7Ra	1: 10
16	488nm	BB515	Tim3	1: 80
17		BB630	BTLA	1: 80
18		BB660	CD27	1: 160
19		BB700	CD161	1: 20
20		BB790	CD38	1: 80
21	532nm	PE	Lag3	1: 20
22		PE-CF594	TCRgd	1: 20
23		PE-Cy5	CD137	1: 20
24		PE-Cy5.5	CD19	1: 160
25		PE-Cy7	CCR7	1: 40
26	628nm	eFlour660	*KI67*	1: *1000*
27		AF700	*GrzmB*	*1: 80*
28		APC-H7	CD4	1: 40

PANEL 3: Transcription factor panel

		Fluorophore	Antigen	Dilutions	
1	355nm	BUV395	CD8	1: 80	
2		L/D UV Blue	dead	1: 500	
3		BUV496	CD3	1: 40	
4		BUV563	CD25	1: 40	
5		BUV661	TCRgd	1: 40	
6		BUV737	CD69	1: 80	
7		BUV805	CD45	1: 80	
8	405nm	BV421	MR1-Tet	1: 500	
9		BV510	CCR7	1: 10	
10		BV570	CD45RO	1: 20	
11		BV605	CD39	1: 20	
12		BV650	Tim3	1: 20	
13		BV711	CCR5	1: 20	
14		BV750	CD103	1: 160	
15		BV786	*KI-67*	1: 320	*ic*
16	488nm	BB515	CD45RA	1: 40	
17		BB630	*CTLA-4*	1: 80	*ic*
18		BB660	CD127	1: 40	
19		BB700	PD1	1: 20	
20		BB790	TIGIT	1: 80	
21	532nm	PE	*TCF1*	1: 40	*ic*
22		PEeFlour610	*EOMES*	1: 10	*ic*
23		PE-Cy5	CD137	1: 20	
24		PE-Cy5.5	*Foxp3*	1: 10	*ic*
25		PE-Cy7	*Tbet*	1: 20	*ic*
26	628nm	APC	*Tox*	1: *80*	*ic*
27		AF700	*GrzmB*	*1: 80*	*ic*
28		APC-H7	*CD4*	1: 40	

PANEL 4: IL1R1/chemokine panel

		Fluorophore	Antigen	Dilutions
1	355nm	BUV395	CD8	1: 80
2		L/D UV Blue	L/D	1: 500
3		BUV496	CD3	1: 40
4		BUV563	CXCR6	1: 20
5		BUV661	CCR7	1: 80
6		BUV737	ICOS	1: 20
7		BUV805	CD45	1: 80
8	405nm	BV421	CD25	1: 40
9		BV480	CD28	1: 40
10		BV570	CD45RA	1: 160
11		BV605	CD39	1: 40
12		BV650	CD69	1: 20
13		BV711	CCR8	1: 20
14		BV750	CD103	1: 160
15		BV785	CCR5	1: 20
16	488nm	BB515	Tim3	1: 80
17		BB630	BTLA	1: 80
18		BB660	CD127	1: 20
19		BB700	PD1	1: 20
20		BB790	TIGIT	1: 40
21	532nm	PE	IL-1R1	1: 20
22		PE-CF594	CXCR3	1: 20
23		PE-Cy5	CD137	1: 20
24		PE-Cy5.5	CD19	1: 160
25		PE-Cy7	IL18R1	1: 40
26	628nm	APC	IL-1R2	1: *20*
27		AF700	HLA-DR	*1: 160*
28		APC-H7	CD4	1: 40

PANEL 5: Merged IL1R1 and TF panel

		Fluorophore	Antigen	Dilutions	
1	355nm	BUV395	CD8	1: 80	
2		L/D UV Blue	dead	1: 500	
3		BUV496	CD3	1: 40	
4		BUV563	CD56	1: 160	
5		BUV661	CCR7	1: 40	
6		BUV737	ICOS	1: 20	
7		BUV805	CD45	1: 80	
8	405nm	BV421	CD25	1: 40	
9		BV510	*GrzmB*	*1: 20*	*ic*
10		BV570	CD45RA	1: 80	
11		BV605	CD39	1: 40	
12		BV650	CD69	1: 20	
13		BV711	CD14	1: 80	
14		BV750	CD103	1: 160	
15		BV785	*KI67*	*1: 160*	*ic*
16	488nm	BB515	Tim3	1: 80	
17		BB630	*CTLA4*	*1: 80*	*ic*
18		BB660	CD127	1: 40	
19		BB700	PD1	1: 20	
20		BB790	TIGIT	1: 40	
21	532nm	PE	IL1R1	1: 20	
22		PEeFlour610	*EOMES*	*1: 20*	*ic*
23		PE-Cy5	CD137	1: 20	
24		PE-Cy5.5	*Foxp3*	*1: 20*	*ic*
25		PE-Cy7	*Tbet*	*1: 40*	*ic*
26	628nm	APC	*TOX*	*1: 80*	*ic*
27		AF700	*TCF1*	*1: 10*	*ic*
28		APC-H7	CD4	1: 40	

PANEL 6: *In vitro*/suppression assays

		Fluorophore	Antigen	Dilutions
1	355nm	BUV395	CD8	1: 80
2		L/D UV Blue		1:
3		BUV496		1:
4		BUV563		
5		BUV661	CD3	1: 40
6		BUV737	ICOS	1: 40
7		BUV805	(CD45)	1:
8	405nm	CTV	CTV	N.A
9		BV510		1:
10		BV570		1:
11		BV605		1:
12		BV650	CD69	1: 40
13		BV711	OX40	1: 40
14		BV750		1:
15		BV785	CD127	1: 20
16	488nm	BB515	CD25	1: 40
17		BB630	BTLA	1: 80
18		BB660		1:
19		BB700	PD1	1: 20
20		BB790		
21	532nm	PE	IL-1R1	1: 20
22		PE-CF594		1:
23		PE-Cy5	CD137	1: 20
24		PE-Cy5.5		1:
25		PE-Cy7		
26	628nm	APC	L/DFarRed	1: *500*
27		AF700		1:
28		APC-H7	CD4	1: 40

Supplementary Table 3

List of antibodies utilized in the presented study

Reagent	Company	Cat.no/Identifier
CD183 (CXCR3)-PE-CF594 (clone 1C6)	BD Biosciences	Cat#562451; RRID:AB_11153118
CD3-BUV496 (clone UCHT1)	BD Biosciences	Cat#564809; RRID:AB_2744388
CD3-BUV661 (clone UCHT1)	BD Biosciences	Cat#612964; RRID:AB_2870239
CD25-BV421 (clone 2A3)	BD Biosciences	Cat#564033; RRID:AB_2738555
CD25-BUV563 (clone 2A3)	BD Biosciences	Cat#612918; RRID:AB_2870203
HLA-DR-BUV661 (cloneG46-6)	BD Biosciences	Cat#612980; RRID:AB_2870252
ICOS-BUV737 (clone DX29)	BD Biosciences	Cat#749665; RRID:AB_2873929
CD8-BUV395 (clone RPA-T8)	BD Biosciences	Cat#563795; RRID:AB_2722501
TCRgd-PE-CF594 (clone B1)	BD Biosciences	Cat#562511; RRID:AB_2737631
CD14-BV570 (clone M5E2)	BioLegend	Cat#301832; RRID:AB_2563629
PD1-BB700 (clone EH12.1)	BD Biosciences	Cat#566460; RRID:AB_2744348
PD1-BV605 (clone EH12.1)	BD Biosciences	Cat#563245; RRID:AB_2738091
CD69-BV650 (clone FN50)	BD Biosciences	Cat#563835; RRID:AB_2738442
CD45RA-BV570 (clone HI100)	BioLegend	Cat#304132; RRID:AB_2563813
CD103-BV750 (clone Ber-ACT8)	BD Biosciences	Cat#747099; RRID:AB_2871852
CD127-Biotin (A019D5)	BioLegend	Cat#351346; RRID:AB_2566509
CD127-BV786 (clone HIL-7R-M21)	BD Biosciences	Cat#563324; RRID:AB_2738138
Tim3-BB515 (clone 7D3)	BD Biosciences	Cat#565568; RRID:AB_2744368
CD16-BUV496 (clone 3G8)	BD Biosciences	Cat#612944; RRID:AB_2870224
CD27-BB660 (clone M-T271)	BD Biosciences, custom	Cat#624295
CD161-BB700 (clone DX12)	BD Biosciences	Cat#745791; RRID:AB_2743247
CD38-BB790 (clone HIT2)	BD Biosciences, custom	Cat#624296
CD39-BV605 (clone A1)	BioLegend	Cat#328236; RRID:AB_2750430
CD137-PECy5 (clone 4B4-1)	BD Biosciences	Cat#551137; RRID:AB_394067
CD19-PE-Cy5.5 (clone SJ25-C1)	Thermo Fisher	Cat#MHCD1918; RRID:AB_1465597
CD197 (CCR7)-BUV661 (clone 2-L1-A)	BD Biosciences	Cat#749824; RRID:AB_2874072
CD197 (CCR7)-PE-Cy7 (clone 3D12)	BD Biosciences	Cat#557648; RRID:AB_396765
CD4-APCH7 (clone RPA-T4)	BD Biosciences	Cat#560158; RRID:AB_1645478
CD40-BUV395 (clone 5C3)	BD Biosciences	Cat#565202; RRID:AB_2739110
CD56-BUV563 (clone NCAM16.2)	BD Biosciences	Cat#612928; RRID:AB_2870213
CD86-BUV737 (clone FUN-1)	BD Biosciences	Cat#612784; RRID:AB_2814790
CX3CR1-PE-Cy7 (clone 2A9-1)	BioLegend	Cat#341612; RRID:AB_10900816
CD28-BV480 (clone CD28.2)	BD Biosciences	Cat#566110; RRID:AB_2739512
CD141-BV605 (clone 1A4)	BD Biosciences	Cat#740421; RRID:AB_2740151
Sirpa-BV650 (clone SE5A5)	BD Biosciences	Cat#743565; RRID:AB_2741588
OX40-BV711 (clone Ber-ACT35)	BioLegend	Cat#350029; RRID:AB_2632863
CD11b-BV750 (clone ICRF44)	BD Biosciences, custom	Cat#747357; RRID:AB_2872054
CD123-BV786 (clone 7G3)	BD Biosciences	Cat#564196; RRID:AB_2738662
CD206-BB515 (clone 19.2)	BD Biosciences	Cat#564668; RRID:AB_2738882
CD32-BB700 (clone FLI8.26)	BD Biosciences	Cat#742216; RRID:AB_2871430
Lag3-PE (clone T47-530)	BD Biosciences	Cat#565617
CD163-PECF594 (clone GHI/61)	BD Biosciences	Cat#562670; RRID:AB_2737711
CD80-PECy5 (clone L307.4)	BD Biosciences	Cat#559370; RRID:AB_397239
CD1c-AF647 (clone F10/21A3)	BD Biosciences	Cat#565048; RRID:AB_2744318
CD11c-AF700 (clone B-ly6)	BD Biosciences	Cat#561352; RRID:AB_10612006
HLA-DR-APC-H7 (clone G46-6)	BD Biosciences	Cat#561358; RRID:AB_10611876
HLA-DR-APC-R700 (clone G46-6)	BD Biosciences	Cat#565127; RRID:AB_2732055
CXCR6-BUV563 (clone 13B 1E5)	BD Biosciences	Cat#748450; RRID:AB_2872866
CD45-BUV805 (HI30)	BD Biosciences	Cat#612891; RRID:AB_2870179
CCR8-BV711 (clone 433H)	BD Biosciences	Cat#747575; RRID:AB_2744146
CD195-BV786 (clone 3A9)	BD Biosciences	Cat#565001; RRID:AB_2739039
CD272-BB630-P (clone J168-540)	BD Biosciences, custom	Cat#624294
TIGIT-BB790 (clone 741182)	BD Biosciences, custom	Cat#624452
Streptavidin-BB660-P2	BD Biosciences, custom	Cat#624295
IL-1R1-PE (polyclonal)	R&D Systems	Cat#FAB269P-100; RRID:AB_2124912
IL-18R1α-PECy7 (clone H44)	BioLegend	Cat#313812; RRID:AB_2800827
IL-1R2-APC (clone 34141)	R&D Systems	Cat#FAB663A; RRID:AB_10569839
CD273-BV421 (clone MIH18)	BD Biosciences	Cat#563842; RRID:AB_2738445
CD85k-BV480 (clone ZM3.8)	BD Biosciences	Cat#746718; RRID:AB_2743983
CD68-BV711 (cloneY1/82A)	BD Biosciences	Cat#565594; RRID:AB_2739297
CD274-BB660-P (clone MIH1)	BD Biosciences, custom	Cat#624295
Axl-PE (clone 108724)	R&D Systems	Cat#FAB154P
KI67-eFluor 660 (clone SolA15)	ThermoFisher Scientific	Cat#50-5698-80; RRID:AB_2574234
Granzyme B AF700 (clone QA16A02)	BioLegend	Cat#372222; RRID:AB_2728389
TCF1-PE	Cell Signaling	Cat#14456S; RRID:AB_2798483
TCF1-AF700	Cell Signaling	Cat#90904S;
TOX-APC	Miltenyi	Cat#130-118-335; RRID:AB_2751485
CCR7-BUV661	BD Biosciences	Cat#749824; RRID:AB_2874072
CTLA4-BB630	BD Biosciences, custom	Cat#624294; RRID:AB_11042699

Tbet-PECy7	Thermo Fisher Scientific	Cat#25-5825-82;
Human MR1 5-OP-RU BV421	NIH Tetramer Facility	N.A
Human TruStain FcX (Fc-Block)	BioLegend	Cat#422302; RRID:AB_2818986
CD3-Ab-O (clone SK7)	BD Biosciences	Cat#940000; RRID:AB_2875891
CD4-Ab-O (clone SK3)	BD Biosciences	Cat#940001; RRID:AB_2875892
CD8-Ab-O (clone RPA-T8)	BD Biosciences	Cat#940003; RRID:AB_2875894
CD19-Ab-O (clone SJ25C1)	BD Biosciences	Cat#940004; RRID:AB_2875895
CD14-Ab-O (clone MϕP9)	BD Biosciences	Cat#940005; RRID:AB_2875896
CD16-Ab-O (clone 3G8)	BD Biosciences	Cat#940006; RRID:AB_2875897
CD56-Ab-O (clone NCAM16.2)	BD Biosciences	Cat#940007; RRID:AB_2875898
CD11b-Ab-O (clone M1/70)	BD Biosciences	Cat#940008; RRID:AB_2875899
CD25-Ab-O (clone 2A3)	BD Biosciences	Cat#940009; RRID:AB_2875900
HLA-DR-Ab-O (cloneG46-6)	BD Biosciences	Cat#940010; RRID:AB_2875901
CD45RA-Ab-O (clone HI100)	BD Biosciences	Cat#940011; RRID:AB_2875902
CD127-Ab-O (clone HIL-7R-M21)	BD Biosciences	Cat#940012; RRID:AB_2875903
CD38-Ab-O (clone HIT2)	BD Biosciences	Cat#940013; RRID:AB_2875904
CD279-Ab-O (clone EH12.1)	BD Biosciences	Cat#940015; RRID:AB_2875906
CD28-Ab-O (clone CD28.2)	BD Biosciences	Cat#940017; RRID:AB_2875908
CD27-Ab-O (clone M-T271)	BD Biosciences	Cat#940018; RRID:AB_2875909
CD69-Ab-O (clone FN 50)	BD Biosciences	Cat#940019; RRID:AB_2875910
CD123-Ab-O (clone 7G3)	BD Biosciences	Cat#940020; RRID:AB_2875911
CD45RO-Ab-O (clone UCHL1)	BD Biosciences	Cat#940022; RRID:AB_2875913
CD11c-Ab-O (clone B-Ly6)	BD Biosciences	Cat#940024; RRID:AB_2875915
CD86-Ab-O (clone FUN-1)	BD Biosciences	Cat#940025; RRID:AB_2875916
CD183-Ab-O (clone 1C6/CXCR3)	BD Biosciences	Cat#940030; RRID:AB_2875921
CD196-Ab-O (clone 11A9)	BD Biosciences	Cat#940033; RRID:AB_2875924
CD80-Ab-O (clone L307.4)	BD Biosciences	Cat#940036; RRID:AB_2875927
CD278-Ab-O (clone DX29)	BD Biosciences	Cat#940043; RRID:AB_2875934
CD194-Ab-O (clone 1G1)	BD Biosciences	Cat#940047; RRID:AB_2875938
CD40-Ab-O (clone 5C3)	BD Biosciences	Cat#940049; RRID:AB_2875940
CD137-Ab-O (clone 4B4-1)	BD Biosciences	Cat#940055; RRID:AB_2875946
TCRgd-Ab-O (clone B1)	BD Biosciences	Cat#940057; RRID:AB_2875948
CD163-Ab-O (clone GH1/61)	BD Biosciences	Cat#940058; RRID:AB_2875949
CD134-Ab-O (clone ACT35)	BD Biosciences	Cat#940060; RRID:AB_2875951
Tim3-Ab-O (clone 7D3)	BD Biosciences	Cat#940066; RRID:AB_2875957
CD103-Ab-O (clone Ber-ACT8)	BD Biosciences	Cat#940067; RRID:AB_2875958
CD206-Ab-O (clone 19.2)	BD Biosciences	Cat#940068; RRID:AB_2875959
CD32-Ab-O (clone FLI8.26)	BD Biosciences	Cat#940069; RRID:AB_2875960
CD161-Ab-O (clone DX12)	BD Biosciences	Cat#940070; RRID:AB_2875961
CD39-Ab-O (clone TU66)	BD Biosciences	Cat#970073; RRID:AB_2875964
CD141-Ab-O (clone 1A4)	BD Biosciences	Cat#940079; RRID:AB_2875970
Lag3-Ab-O (clone T47-530)	BD Biosciences	Cat#940080; RRID:AB_2875971
CD1c-Ab-O (clone F10/21A3)	BD Biosciences	Cat#940083; RRID:AB_2875974
CD244-Ab-O (clone 2-69)	BD Biosciences	Cat#940362; RRID:AB_2876232
CD274-Ab-O (clone B7-H1)	BD Biosciences	Cat#940035; RRID:AB_2875926
CX3CR1-Ab-O (clone 2A9-1)	BD Biosciences	Cat#940216; RRID:AB_2876098
VISTA-Ab-O (clone MIH65.rMAb)	BD Biosciences	Cat#940497; RRID:AB_2876339
CCR7-Ab-O (clone 2-L1-A)	BD Biosciences	Cat#940394; RRID:AB_2876258
CD5-Ab-O (clone UCHT2)	BD Biosciences	Cat#940038; RRID:AB_2875929
CD195-Ab-O (clone2D7/CCR5)	BD Biosciences	Cat#940050; RRID:AB_2875941
CXCR6-Ab-O (clone 13B 1E5)	BD Biosciences	Cat#940234; RRID:AB_2876115
CCR2-Ab-O (clone LS132.1D9)	BD Biosciences	Cat#940286; RRID:AB_2876163
Anti-PE-Ab-O (E31-1459) (used to detect IL-1R1-PE antibody in AbSeq experiment)	BD Biosciences (custom)	Cat#2216Z / 120162

Supplementary Table 4

Custom gene panel for Rhapsody experiments (data shown in Figure 4)

For genes in the base panel, please refer to the "Human Immune Response Panel"

(BD Biosciences, #633750)

1	ADAM15		33	FCGR3B		65	MMP2
2	ADAM17		34	FOS		66	MNDA
3	ADAM28		35	FOXO3		67	MRC1
4	AHR		36	GATA3		68	MX1
5	AIF1		37	GZMM		69	MX2
6	ALCAM		38	HIF1A		70	MXRA8
7	AXL		39	HLA-DPB1		71	NLRP3
8	BATF		40	HLA-DQA1		72	PDCD1LG2
9	BIRC5		41	HOPX		73	PTPN6
10	C1ORF54		42	ICOSLG		74	S100A8
11	CAPG		43	ID2		75	S1PR1
12	CCL3L3		44	ID3		76	SBK1
13	CCL4L2		45	IFITM1		77	SERPINA1
14	CCR6		46	IFNGR2		78	SOCS3
15	CD200R		47	IFNL1		79	STK38
16	CD207		48	IL10		80	TGFBR2
17	CD40LG		49	IL10RB		81	TIMP1
18	CD68		50	IL18BP		82	TIMP2
19	CD83		51	IL23A		83	TIMP3
20	CDH1		52	IL27		84	TMEM123
21	CLEC9A		53	IL6R		85	TNFRSF14
22	CLU		54	IL6ST		86	TNFRSF18
23	CRTAM		55	IRF7		87	TNFRSF1B
24	CSF1		56	ITGA1		88	TNFSF12
25	CSF1R		57	ITGA5		89	TOX
26	CSF2RA		58	KLF2		90	TREM2
27	CTSK		59	KLF3		91	TSPAN18
28	CTSS		60	KLF6		92	VCAN
29	DUSP6		61	KLRC2		93	VEGFB
30	EBI3		62	KLRD1		94	XCL1
31	FBLN2		63	LYZ		95	XCL2
32	FBN2		64	MMP1		96	ZFP36
