# Supplementary material for: Extricating human tumour immune alterations from tissue inflammation
Source: Nature. 2022 May 11;605(7911):728–35. doi: 10.1038/s41586-022-04718-w (PMC9132772; doi:10.1038/s41586-022-04718-w)
Supplement: Supplementary file 2 — Reporting Summary [file 41586_2022_4718_MOESM2_ESM.pdf]

nature research

Corresponding author(s): Martin Prlic
Last updated by author(s): February 15th 2022

Reporting Summary

Nature Research wishes to improve the reproducibility of the work that we publish. This form provides structure for consistency and transparency in reporting. For further information on Nature Research policies, see our Editorial Policies and the Editorial Policy Checklist.

Statistics

For all statistical analyses, confirm that the following items are present in the figure legend, table legend, main text, or Methods section.

n/a	Confirmed	
☐	☒	The exact sample size (*n*) for each experimental group/condition, given as a discrete number and unit of measurement
☐	☒	A statement on whether measurements were taken from distinct samples or whether the same sample was measured repeatedly
☐	☒	The statistical test(s) used AND whether they are one- or two-sided<br>*Only common tests should be described solely by name; describe more complex techniques in the Methods section.*
☒	☐	A description of all covariates tested
☐	☒	A description of any assumptions or corrections, such as tests of normality and adjustment for multiple comparisons
☐	☒	A full description of the statistical parameters including central tendency (e.g. means) or other basic estimates (e.g. regression coefficient) AND variation (e.g. standard deviation) or associated estimates of uncertainty (e.g. confidence intervals)
☐	☒	For null hypothesis testing, the test statistic (e.g. *F*, *t*, *r*) with confidence intervals, effect sizes, degrees of freedom and *P* value noted<br>*Give P values as exact values whenever suitable.*
☒	☐	For Bayesian analysis, information on the choice of priors and Markov chain Monte Carlo settings
☒	☐	For hierarchical and complex designs, identification of the appropriate level for tests and full reporting of outcomes
☒	☐	Estimates of effect sizes (e.g. Cohen's *d*, Pearson's *r*), indicating how they were calculated

Our web collection on statistics for biologists contains articles on many of the points above.

Software and code

Policy information about availability of computer code

Data collection	FACSDiva (BD Biosciences), cellranger and cellranger vdj (10x Genomics), BD Rhapsody preprocessing pipeline (www.sevenbridges.com)
Data analysis	FlowJo v10.7.x (BD Biosciences), R v3.5.x and v3.6.x (R Project for Statistical Computing). For bulk RNA-seq data: STAR aligner (v2.4.2a), HTSeq-count (v0.4.1), PICARD (v1.134), FASTQC (v0.11.3), Samtools (v1.2), HTSeq-count (v0.4.1). For scRNA-seq data: Seurat v3.x and associated packages (www.satijalab.org), CellRanger (v3.0.x) and Loupe VDJ Browser (v3.0, 10x Genomics), NicheNet (v0.1.0), Harmony (v1.0), Premessa (R package v0.2.4). Other: FAUST (R package, v0.5.x), Microsoft Excel (v16.57), Prism v9.x (GraphPad).<br>Custom workflows used in this manuscript are available on https://github.com/MairFlo/Tumor_vs_Inflamed and https://github.com/akonecny/Bulk-RNAseq-Tumor_vs_Inflamed

For manuscripts utilizing custom algorithms or software that are central to the research but not yet described in published literature, software must be made available to editors and reviewers. We strongly encourage code deposition in a community repository (e.g. GitHub). See the Nature Research guidelines for submitting code & software for further information.

Data

Policy information about availability of data

All manuscripts must include a data availability statement. This statement should provide the following information, where applicable:

- Accession codes, unique identifiers, or web links for publicly available datasets
- A list of figures that have associated raw data
- A description of any restrictions on data availability

The single-cell sequencing data as well as the bulk RNA sequencing data discussed in this publication have been deposited in the NCBI's Omnibus database (https://www.ncbi.nlm.nih.gov/geo/) at GEO-ID GSE163633. Alignment was based on the GRCh38 reference genome. Flow cytometry raw data have been deposited at www.flowrepository.org using the Identifiers FR-FCM-Z4UX, FR-FCM-Z4UP and FR-FCM-Z4UQ or can be requested from the first author.

Field-specific reporting

Please select the one below that is the best fit for your research. If you are not sure, read the appropriate sections before making your selection.

☒ Life sciences ☐ Behavioural & social sciences ☐ Ecological, evolutionary & environmental sciences

For a reference copy of the document with all sections, see nature.com/documents/nr-reporting-summary-flat.pdf

Life sciences study design

All studies must disclose on these points even when the disclosure is negative.

Sample size	Total sample size of data shown in paper is n=81 donors
Data exclusions	Data was only excluded if technical issues during processing, flow cytometry staining or acquisition were found (based on comparison to our technical reference sample, see material and methods section)
Replication	N.A. Technical replicates were not possible with these human tissues, but technical controls were included.
Randomization	N.A. (no clinical trial)
Blinding	N.A. (tumor tissues and inflamed oral tissues came from different sources making blinding impossible)

Reporting for specific materials, systems and methods

We require information from authors about some types of materials, experimental systems and methods used in many studies. Here, indicate whether each material, system or method listed is relevant to your study. If you are not sure if a list item applies to your research, read the appropriate section before selecting a response.

Materials & experimental systems

n/a	Involved in the study	
☐	☒	Antibodies
☐	☒	Eukaryotic cell lines
☒	☐	Palaeontology and archaeology
☐	☒	Animals and other organisms
☐	☒	Human research participants
☒	☐	Clinical data
☒	☐	Dual use research of concern

Methods

n/a	Involved in the study	
☒	☐	ChIP-seq
☐	☒	Flow cytometry
☒	☐	MRI-based neuroimaging

Antibodies

Antibodies used	All utilized antibodies, including clones, catalogue numbers and dilutions used, are listed in Supplementary table 2.
Validation	All antibodies used in flow cytometry were titrated and assessed for biologically meaningful staining patterns by assessing expression against canonical main immune lineages (CD19+ B cells, CD3+ T cells, CD14+ monocytes etc). Antibodies from BD Biosciences undergo routine QC testing for reactivity (see www.bdbiosciences.com), antibodies from Thermo Fisher Scientific were verified by relative expression assays (see www.thermofisher.com), for antibodies from Biolegend each antibody is quality control tested by immunfluorescent staining with flow cytometric analysis (see www.biolegend.com).

Eukaryotic cell lines

Policy information about cell lines

Cell line source(s)	SCC-15 cells, obtained from ATCC: https://www.atcc.org/products/crl-1623
Authentication	Cells were authenticated by ATCC (certificate analysis LOT 70015828)
Mycoplasma contamination	Cells were tested negative for mycoplasma
Commonly misidentified lines (See ICLAC register)	N.A.

Animals and other organisms

Policy information about studies involving animals; ARRIVE guidelines recommended for reporting animal research

Laboratory animals	Mus musculus Foxp3eGFP-Cre-ERT2 spleens+lymph nodes from male mice, age 8 weeks or older (courtesy of Dr. Jennifer Lund, FHCRC).
Wild animals	N.A.
Field-collected samples	N.A.
Ethics oversight	Mouse protocols were approved by and in compliance with the ethical regulations of Fred Hutchinson Cancer Research Center's IACUC.

Note that full information on the approval of the study protocol must also be provided in the manuscript.

Human research participants

Policy information about studies involving human research participants

Population characteristics	Tumor tissue donors were collected by an independent organization appointed specifically for that task (NWBioSpecimen, Seattle, WA) based on tumor type (HNSCC, papillary carcinoma, breast cancer) and size. Oral mucosal tissues were collected from routine surgeries, and based on the type of procedure (i.e. likely to yield a large enough tissue piece for processing).
Recruitment	N.A. (collection of discarded tissues from surgery, see above)
Ethics oversight	Insitutional Review board (IRB) of the Fred Hutchinson Cancer Research Center (IRB#6007-972 and IRB#8335)

Note that full information on the approval of the study protocol must also be provided in the manuscript.

Flow Cytometry

Plots

Confirm that:

- [x] The axis labels state the marker and fluorochrome used (e.g. CD4-FITC).
- [x] The axis scales are clearly visible. Include numbers along axes only for bottom left plot of group (a 'group' is an analysis of identical markers).
- [x] All plots are contour plots with outliers or pseudocolor plots.
- [x] A numerical value for number of cells or percentage (with statistics) is provided.

Methodology

Sample preparation

After surgical procedures, fresh tissue samples were placed immediately into a 50ml conical tube with complete media (RP10: RPMI1640 supplemented with Penicillin, Streptomcyin and 10% Fetal Bovine Serum (FBS), RP10) and kept at 4°C. Samples were processed within 1-4 hours after collection based on optimized protocols adapted from (Leelatian et al., 2017). Briefly, tissue pieces were minced using a scalpel into small pieces and incubated with Collagenase II (Sigma-Aldrich, 0.7 mg/ml) and DNAse (5 Units/ml) in RPMI1640 with 7.5% FBS for 30-45 minutes depending on sample size. Subsequently, any remaining tissue pieces were mechanically disrupted by repeated resuspension with a 30 ml syringe with a large bore tip (16x1 ½ blunt). The cell suspension was filtered using a 70um cell strainer, washed in RPMI1640 and immediately used for downstream procedures.

Peripheral blood samples (1-10 ml) were collected in ACD tubes and then processed using SepMate tubes (StemCell Technologies, #85450) and Lymphoprep (Stem Cell Technologies, #07851) according to manufacturer protocols. Briefly, whole blood samples were centrifuged for 10 minutes at 400g, and the plasma supernatant was collected separately and immediately frozen at -80°C. Remaining cells were resuspended in 30ml of PBS and pipetted on top of 13.5ml Lymphoprep in a SepMate tube. After centrifugation for 16 minutes at 1200g, the mononuclear cell fraction in the supernatant was poured into a fresh 50ml tube, washed with PBS and immediately used for downstream procedures. For some blood samples from dental surgery patients, red blood cells were lysed using ACK-lysis buffer, and the remaining white blood cells were directly used for downstream staining.
If required, cells isolated from tissue samples or from peripheral blood were frozen using either a 90%FBS/10%DMSO mixture or Cell Culture Freezing Medium (Gibco, #12648010), and stored in liquid nitrogen until used for downstream procedures.

For flow cytometric analysis good practices were followed as outlined in the guidelines for use of flow cytometry (Cossarizza et al, 2021). Directly following isolation/thawing, cells were incubated with Fc-blocking reagent (BioLegend Trustain FcX, #422302) and fixable UV Blue Live/Dead reagent (ThermoFisher, #L34961) in PBS (Gibco, #14190250) for 15 minutes at room temperature. After this, cells were incubated for 20 minutes at room temperature with 50 [illegible]l total volume of antibody master mix freshly prepared in Brilliant staining buffer (BD Bioscience, #563794), followed by two washes. All antibodies were titrated and used at optimal dilution, and staining procedures were performed in 96-well round-bottom plates (for cell sorting in 5ml polystyrene tubes). A detailed list of the main panels used, including fluorochromes, antibody catalogue numbers and final dilutions is provided in Suppl. Table 2 (panels designed according to best practices as described in Mair et

al, 2019). For sorting, cells were immediately used after staining, and for analysis, the stained cells were fixed with 4% PFA (Cytofix/Cytoperm, BD Biosciences) for 20 minutes at room temperature, washed, resuspended in FACS buffer and stored at 4C in the dark until acquisition. If necessary, intracellular (CD68, Granzyme B, CTLA-4) or intranuclear staining (Foxp3, KI67, TCF1, T-bet, EOMES) was performed following the appropriate manufacturer protocols (eBioscience Foxp3/Transcription Factor Staining Buffer Set, Thermo Fisher #00-5532-00)
Single-stained controls were prepared with every experiment using antibody capture beads (BD Biosciences anti-mouse, #552843 or anti-mouse Plus, and anti-rat, #552844) diluted in FACS buffer, or cells for Live/Dead reagent, and treated exactly the same as the samples (including fixation procedures). For each staining of experimental samples, a PBMC sample from the same healthy donor was stained with the same panel as a longitudinal reference control (data not shown).

All samples were acquired using a FACSymphony A5 (BD Biosciences), equipped with 30 detectors and 355nm (65mW), 405nm (200mW), 488nm (200mW), 532nm (200mW) and 628nm (200mW) lasers and FACSDiva acquisition software (BD Biosciences). Full details on the optical configuration of the instruments used are provided in Mair et al, 2018 23. Detector voltages were optimized using a modified voltage titration approach 60 and standardized from day to day using MFI target values and 6-peak Ultra Rainbow Beads (Spherotec, # URCP-38-2K) 59. After acquisition, data was exported in FCS 3.1 format and analyzed using FlowJo (version 10.6.x, and 10.7.x, BD Biosciences). Samples were analyzed using a combination of manual gating and computational analyses approaches, with doublets being excluded by FSC-A vs FSC-H gating. For samples acquired on different experimental days with the T cell or APC panel, files were exported as compensated data and analyzed combined together in a new workspace. Gates were kept the same across all samples except where changes in the density distribution clearly indicated the need for sample-specific adjustment. For the APC panel, PD-L1 (V450 channel) as well as CD85k (V510 channel) were excluded from analysis because of interference/high variability from highly auto-fluorescent myeloid cells in some samples. For the T cell panel, Granzyme-B staining showed donor-specific shifts in intensity, requiring sample-specific adjustments of gates.

Details of the flow cytometry staining procedures and antibodies used are described in the material and methods section and in all panels are listed in Supplementary Table 2.

Instrument

FACSymphony A5 (BD Biosciences), FACSymphony S6 (BD Biosciences) and FACSAria III (BD Biosciences). Laser configuration for the analyzers is listed in material and methods, and full optical configuration is listed in Mair et al, Cytometry Part A 2018.

Software

BD FACSDiva (BD Biosciences) and FlowJo v10.6.x and v10.7.x (BD Biosciences)

Cell population abundance

For cell sorting, an aliquot of cells was taken immediatetely after sorting and re-analyzed at the same instrument. A representative analysis is shown in Extended data figure 4 and in main Figure 4.

Gating strategy

Gating strategies used for analytical samples are shown in Extended data figure 1 and 2. Gating strategies used for sorting experiments are shown in Extended data figure 4, including the FSC-SSC gate. Gate boundaries were set either based on control samples (fluorescence minus-one or flourescence-minus-two controls), or followed density distributions based on best practices.

☒ Tick this box to confirm that a figure exemplifying the gating strategy is provided in the Supplementary Information.
